# Natural Transformation in *Deinococcus radiodurans*: A Genetic Analysis Reveals the Major Roles of DprA, DdrB, RecA, RecF, and RecO Proteins

**DOI:** 10.3389/fmicb.2020.01253

**Published:** 2020-06-18

**Authors:** Solenne Ithurbide, Geneviève Coste, Johnny Lisboa, Nicolas Eugénie, Esma Bentchikou, Claire Bouthier de la Tour, Dominique Liger, Fabrice Confalonieri, Suzanne Sommer, Sophie Quevillon-Cheruel, Pascale Servant

**Affiliations:** Université Paris-Saclay, CEA, CNRS, Institute for Integrative Biology of the Cell (I2BC), Gif-sur-Yvette, France

**Keywords:** *Deinococcus radiodurans*, natural transformation, DNA uptake, homologous recombination, DprA, RecA, RecFOR, DdrB

## Abstract

Horizontal gene transfer is a major driver of bacterial evolution and adaptation to environmental stresses, occurring notably via transformation of naturally competent organisms. The *Deinococcus radiodurans* bacterium, characterized by its extreme radioresistance, is also naturally competent. Here, we investigated the role of *D. radiodurans* players involved in different steps of natural transformation. First, we identified the factors (PilQ, PilD, type IV pilins, PilB, PilT, ComEC-ComEA, and ComF) involved in DNA uptake and DNA translocation across the external and cytoplasmic membranes and showed that the DNA-uptake machinery is similar to that described in the Gram negative bacterium *Vibrio cholerae*. Then, we studied the involvement of recombination and DNA repair proteins, RecA, RecF, RecO, DprA, and DdrB into the DNA processing steps of *D. radiodurans* transformation by plasmid and genomic DNA. The transformation frequency of the cells devoid of DprA, a highly conserved protein among competent species, strongly decreased but was not completely abolished whereas it was completely abolished in Δ*dprA* Δ*recF*, Δ*dprA* Δ*recO*, and Δ*dprA* Δ*ddrB* double mutants. We propose that RecF and RecO, belonging to the recombination mediator complex, and DdrB, a specific deinococcal DNA binding protein, can replace a function played by DprA, or alternatively, act at a different step of recombination with DprA. We also demonstrated that a Δ*dprA* mutant is as resistant as wild type to various doses of γ-irradiation, suggesting that DprA, and potentially transformation, do not play a major role in *D. radiodurans* radioresistance.

## Introduction

Natural transformation is a mode of horizontal gene transfer which contributes to the acquisition of new properties and thus to genome evolution. Since the discovery of natural transformation of *Streptococcus pneumoniae* (Griffith, 1928), more than 85 species have now been shown to be naturally transformable (Johnston et al., 2014). This mechanism can be divided into three steps:

1. External double-stranded DNA (dsDNA) capture followed by the translocation of single-stranded DNA (ssDNA) into the cytosol. In most transformable bacteria (except for *Helicobacter pylori*), dsDNA capture is dependent on type II secretion or type IV pilus system proteins (Kruger and Stingl, 2011; Johnston et al., 2014; Matthey and Blokesch, 2016). The translocation of ssDNA across the cytoplasmic membrane requires three components: the DNA receptor (ComEA) (Seitz et al., 2014), the permease/channel protein (ComEC) (Pimentel and Zhang, 2018), and the ATPase protein (ComF) (Diallo et al., 2017).
2. Protection of the translocated ssDNA from degradation. Single-stranded DNA binding proteins (SSB) protect internalized ssDNA from degradation by nucleases and limit the loading of the RecA recombinase. In *S. pneumoniae*, SsbB directly protects internalized ssDNA and maintains a DNA reservoir in the cell (Attaiech et al., 2011). DprA, a member of the recombination mediator proteins (RMP) family dedicated to natural bacterial transformation, interacts with naked and SSB-coated ssDNA and also protects DNA (Mortier-Barriere et al., 2007; Quevillon-Cheruel et al., 2012).
3. Integration into the chromosome of the incoming DNA by homologous recombination or a reconstitution of an autonomous plasmid. The integration of the internalized ssDNA into the chromosome is catalyzed by the RecA recombinase. Recombination mediators are required to load RecA on SSB-coated ssDNA. DprA was reported in *S. pneumoniae* as being a key partner of RecA, acting as a specific mediator for its loading on the incoming ssDNA (Mortier-Barriere et al., 2007; Quevillon-Cheruel et al., 2012). The establishment of plasmid DNA requires a single strand annealing activity to pair internalized complementary plasmid DNA fragments in order to reconstitute a circular replicon in naturally transformable bacteria such as *S. pneumoniae* and *Bacillus subtilis* (Saunders and Guild, 1981; Kidane et al., 2009).

The *D. radiodurans* bacterium, characterized by its extreme resistance to the lethal effects of ionizing and ultraviolet radiations, has been shown to be naturally transformable (Moseley and Setlow, 1968), a property that contributed to the development of this species as a model organism. However, discovering the proteins playing an important role in *D. radiodurans* natural transformation has never been a central question in the field. Many studies, focusing on *D. radiodurans* radioresistance, revealed that its extreme resistance to DNA damaging agents is correlated with its ability to reconstruct a functional genome from hundreds of chromosomal fragments, mainly through an extended synthesis-dependent strand annealing (ESDSA) pathway and through homologous recombination, two pathways mediated by RecFOR and RecA (Zahradka et al., 2006; Slade et al., 2009; Bentchikou et al., 2010). A RecA-independent single strand annealing (SSA) process also participates in DNA double strand break (DSB) repair and requires DdrB, a *Deinococcus* single stranded DNA binding protein exhibiting a single strand annealing activity (Xu et al., 2010; Bouthier de la Tour et al., 2011; Sugiman-Marangos et al., 2016). Remarkably, it was shown that the cells devoid of DdrB were affected in the establishment of plasmid DNA during natural transformation, a process that requires pairing of internalized plasmid single stranded DNA fragments, whereas they were proficient in transformation by a chromosomal DNA marker that integrates into the host chromosome through homologous recombination (Bouthier de la Tour et al., 2011). This study provided the first evidence linking *Deinococcal* specific DNA repair proteins with natural transformation and raising numerous questions regarding the potential specifics of transformation mechanisms in this species.

Here we identified, by genome analysis, candidate genes described in other bacterial species as being involved in different transformation steps. We focused on genes that might be involved in the protection of the incoming ssDNA and its genome integration or plasmid reconstitution. We showed that, as in other bacteria, DprA plays a key role in DNA transformation and we highlighted the ability of the RecFOR complex and the DdrB protein to partially compensate the absence of DprA in the DNA transformation process.

## Results

### Identification of *D. radiodurans* Genes Involved in DNA Uptake and DNA Translocation During Transformation

Although *D. radiodurans* is classified as a Gram positive bacterium, it was shown that cells contain two membranes (Thompson and Murray, 1981). We identified, by sequence similarity, several *D. radiodurans* genes encoding type IV pilus biogenesis systems, type IV pili, and a fimbrial subunit (Table 1). The *dr0774* gene encodes a protein belonging to the secretin (PilQ) family, which forms, in *V. cholerae*, the entry pore through the outer membrane for the transforming DNA (Seitz and Blokesch, 2014). This protein was found to be one of the most abundant *D. radiodurans* proteins of the inner/outer membrane (Farci et al., 2014). In *D. radiodurans*, the *dr0774* gene is the last gene of an operon encompassing *pilM* (*dr0770*), *pilN* (*dr0771*), *pilO* (*dr0772*), and *pilP* (*dr0773*) genes that encode putative homologs of proteins belonging to an inner membrane alignment subcomplex of type IV pili in *Pseudomonas aeruginosa* and in *V. cholerae* (Burrows, 2012; Tammam et al., 2013). The *dr0548* and *dr1232* genes are homologous to genes encoding type IV pilin in *Deinococcus geothermalis* (Saarimaa et al., 2006). In *Dichelobacter nodosus*, the FimA fimbrial subunit is essential for transformation (Kennan et al., 2001). A homolog of *fimA* was also found in the recent published sequence of *D. radiodurans* R1 (Hua and Hua, 2016). Finally, *dr2065, dr1964*, and *dr1963* are *Deinococcus* gene homologs of *pilD, pilB*, and *pilT*, encoding the PilD peptidase involved in prepilin processing (Marsh and Taylor, 1998) and two traffic ATPases, PilB and PilT, required for DNA uptake and efficient transformation in *V. cholerae* (Seitz and Blokesch, 2013b), respectively.

**Table 1:** *D. radiodurans* genes putatively involved in natural transformation.

**Protein function**	**Protein**	**Gene (White et al., 1999)**	**Gene (Hua and Hua, 2016)**
**The competence pseudopilus**			
Inner membrane subcomplex of type IV pili	*PilM*	*dr0770*	A2G07_09655
Inner membrane subcomplex of type IV pili	*PilN*	*dr0771*	A2G07_09650
Inner membrane subcomplex of type IV pili	*PilO*	*dr0772*	A2G07_09645
Inner membrane subcomplex of type IV pili	*PilP*	*dr0773*	A2G07_09640
Secretin	*PilQ*	*dr0774*	A2G07_09635
Pilin or pseudopilin (type IV)	*Pilin* *pilIV*	*dr0548*	none
Pilin or pseudopilin (type IV)	*Pilin* *pilIV*	*dr1232*	A2G07_07365
Pilin or pseudopilin (type I)	*FimA*	None	A2G07_02580
Prepilin/peptidase	*PilD*	*dr2065*	A2G07_03280
Pilus retraction	*PilT*	*dr1963*	A2G07_03820
Pilus extension	*PilB*	*dr1964*	A2G07_03815
**DNA translocation machinery**			
DNA receptor	*ComEA*	*dr0207*	A2G07_12495
	*ComEA*	*dr1855*	A2G07_04330
Membrane channel	*ComEC*	*dr1854*	A2G07_04335
	*ComEC*	*dr0361*	A2G07_11730
ATP-binding protein	*ComF*	*dr1389*	A2G07_06600
ATP-binding protein	*ComA*	*dr0847*	A2G07_09275
**Processing DNA**			
DprA	*DprA*	*dr0120*	A2G07_12915
Single stranded DNA binding protein	DdrB	dr0070	A2G07_00040
Complex RecFOR	*RecF*	*dr1089*	A2G07_08075
Complex RecFOR	*RecO*	*dr0819*	A2G07_09415
Complex RecFOR	*RecR*	*dr0198*	A2G07_12540
**Homologous recombination**			
Recombinase	*RecA*	*dr2340*	A2G07_01875

We also identified genes encoding the proteins homologous to ComEA (*dr0207* and *dr1855*), ComEC (*dr1854* and *dr0361*), ComA (*dr0847*), and ComF (*dr1389*) proteins constituting a potential DNA translocation machinery similar to that found in Gram negative bacteria (Table 1). ComEA, a predicted membrane-integral protein, shuttles the dsDNA through the periplasm. The transport of DNA across the cytoplasmic membrane is mediated by ComEC (a cytoplasmic membrane channel), potentially in concert with ComF, to introduce ssDNA into the cytoplasm.

To determine whether these proteins may be involved in *D. radiodurans* transformation, we constructed deletion mutants of *dr0774* (*pilQ*)*, dr0548 (pilIV), dr1232 (pilIV), fimA, dr2065* (*pilD*), *dr1963* (*pilT*), *dr1964* (*pilB*), *dr0207* (*comEA*), *dr1855* (*comEA*), *dr1854* (*comEC*), *dr0361* (*comEA*), *dr1389* (*comF*), and *dr0847* (*comA*) genes. Since *D. radiodurans* contains from 4 to 10 genome equivalents, the transformants were purified on selective medium in order to obtain homogenotes containing the deleted allele on all copies of the genome. Homogenotes of these deletion mutants were easily obtained after two cycles of purification on selective medium, and the purity of the strains was verified by PCR. These deletion mutants did not exhibit any significant effect on *D. radiodurans* growth in optimal growth conditions (Table S1), indicating that these genes are not essential for cell viability.

**Figure 1:**
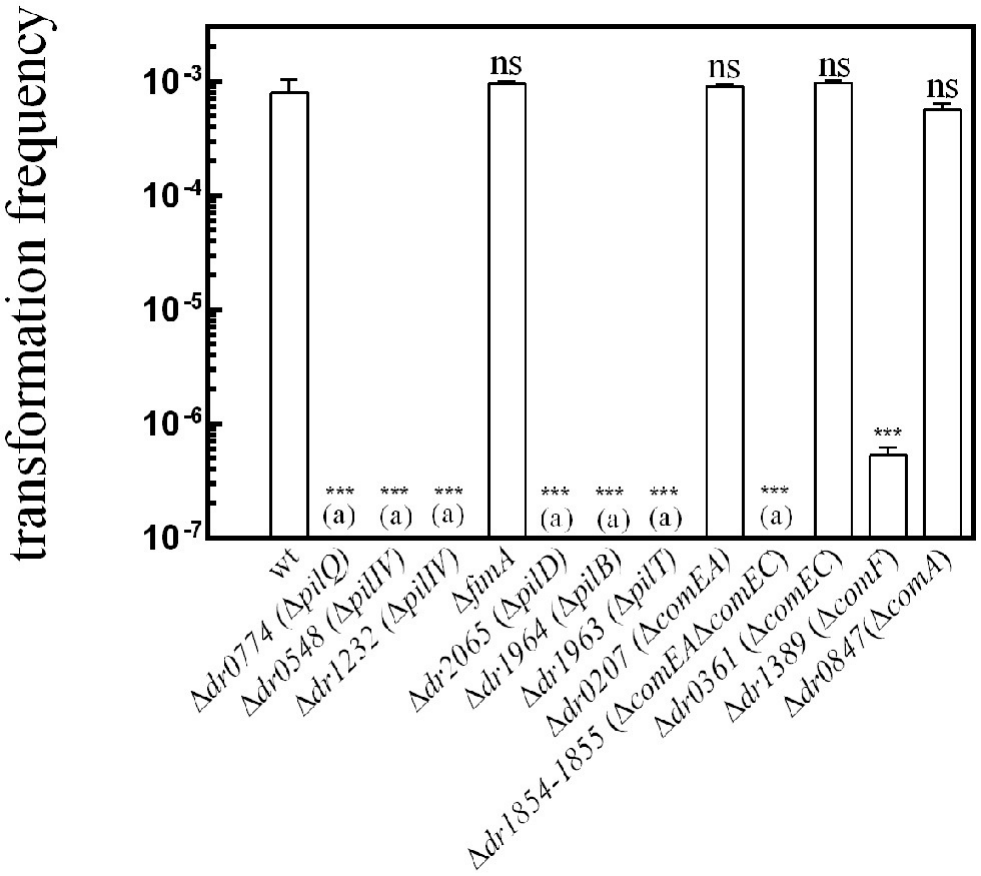
Genes required for the natural transformation machinery of *D. radiodurans*. The putative functions of these genes are listed in Table 1. Cells were transformed with 400 ng of genomic DNA harboring a mutation (Δ9 deletion) in the *rpoB* gene conferring resistance to rifampicin. The results are the average of at least five independent experiments. (a): The frequency of [Rif^R^] transformants is as low as the spontaneous frequency of [Rif^R^] mutants. Statistically significant differences of transformation frequencies of the mutants, compared to those observed in wild type strain, were calculated using the non-parametric Mann Whitney test: ***P < 0.001; ns if *P* > 0.5.

We show here that natural transformation is totally abolished in Δ*dr0774* (*pilQ*), Δ*dr0548 (pilIV)*, Δ*dr1232 (pilIV)*, Δ*dr2065 (pilD)*, Δ*dr1963 (pilT)*, Δ*dr1964 (pilB)*, and Δ*dr1854*Δ*dr1855* (*comEC comEA*) mutant bacteria (Figure 1). The transformation frequency was reduced by a factor of ~2,000 in Δ*dr1389* bacteria devoid of the ComF protein (Figure 1). In contrast, Δ*fimA*, Δ*dr0207* (*comEA)*, Δ*dr0361* (*comEC)*, and Δ*dr0847* (*comA*) mutant bacteria remained proficient for transformation.

To verify that PilD, type IV pilins, PilB, PilT, and ComEC-ComEA are essential for natural transformation and that ComF plays a major role in the transformation process, we performed complementation assays in the mutant strains. We found that expression of the corresponding wild type *pilD* (*dr0548*), *comEC-comEA* (*dr1854*-*dr1855*), and *comF* (*dr1389*) genes from a non-essential ectopic chromosomal locus [*amyE* (*dr1472*)] in the strains devoid of these proteins restored wild-type transformation frequency (Figure S1). Unfortunately, we were not able to obtain strains expressing, *in trans, dr1232*, and *dr0774*.

### RecA Is Essential for Chromosomal Transformation but Not for Plasmid Establishment

**Figure 2:**
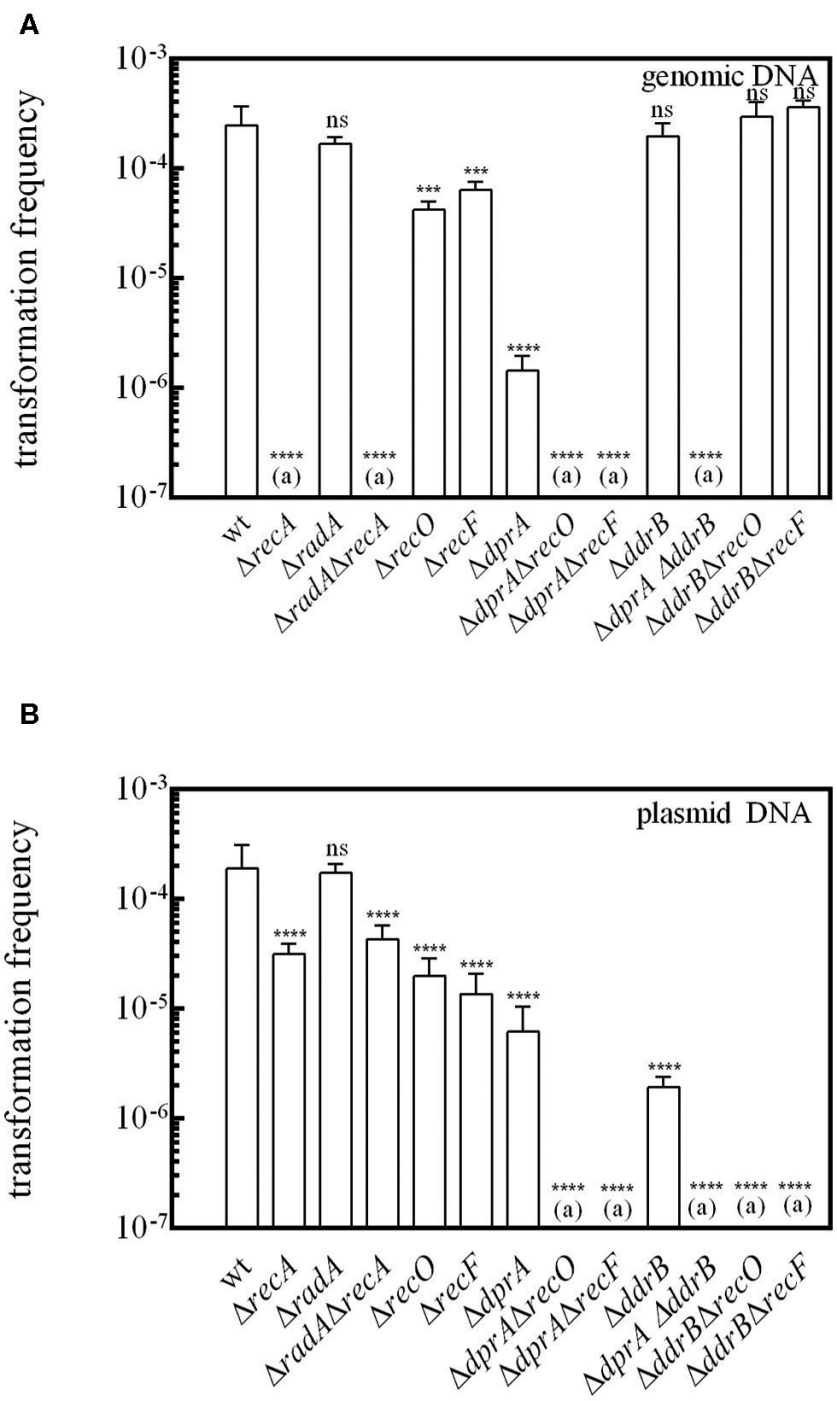
RecO, RecF, and DdrB proteins are required for transformation in cells devoid of DprA. *D. radiodurans* cells were transformed with **(A)** 200 ng genomic DNA from a [Rif^R^] (GY 11733) strain or **(B)** with 200 ng of p11559 plasmid DNA conferring spectinomycin resistance. The results are the average of at least five independent experiments. (a): The frequency of [Rif^R^] or [Spec^R^] transformants is as low as the spontaneous frequency of [Rif^R^] or [Spec^R^] mutants. Statistically significant differences of transformation frequencies of the mutants, compared with those observed in wild type strain, were calculated using the non-parametric Mann Whitney test: ***P < 0.001; ****P < 0.0001; ns if *P* > 0.5.

Transformation of bacteria by genomic DNA requires integration of the incoming DNA into the host chromosome by homologous recombination. Here, we show that, as in other species, the transformation of *D. radiodurans* by genomic DNA is a mechanism totally dependent on the RecA protein (Figure 2A), whereas cells devoid of the RecA protein were only slightly affected in the frequency of transformation by plasmid DNA (5-fold reduction) (Figure 2B). We also measured the frequency of transformation of *D. radiodurans* bacteria devoid of the RadA protein, a conserved homologous recombination effector acting with RecA recombinase to promote ssDNA integration in *S. pneumoniae* (Marie et al., 2017). The Δ*radA* mutant bacteria exhibited the same transformation frequency by genomic and plasmid DNA as the wild type cells (Figure 2), indicating that RadA is not involved in *D. radiodurans* transformation. Moreover, Δ*radA* Δ*recA* bacteria exhibited the same transformation frequency as Δ*recA* mutant bacteria.

The *Deinococcal* RecF, RecO, and RecR recombination mediator proteins, by their ability to load RecA onto its single-stranded DNA substrate, play a crucial role in DNA double strand break repair via ESDSA and recombinational repair pathways (Bentchikou et al., 2010). We show here that the absence of the RecF and RecO proteins reduces the efficiency of transformation by genomic DNA by a factor of 3.6 and 5.4, respectively (Figure 2A), suggesting that other protein(s) are able to promote the loading of RecA on the internalized single-stranded DNA. Expression *in trans* of the *recO, recF*, and *recA* genes from the *amyE* ectopic chromosomal locus in the strains devoid of these proteins fully restored a wild type transformation frequency for genomic DNA (Figure S2). These results confirm the involvement of these three proteins in the transformation process of genomic DNA.

### DprA Plays a Major Role in DNA Transformation

**Figure 3:**
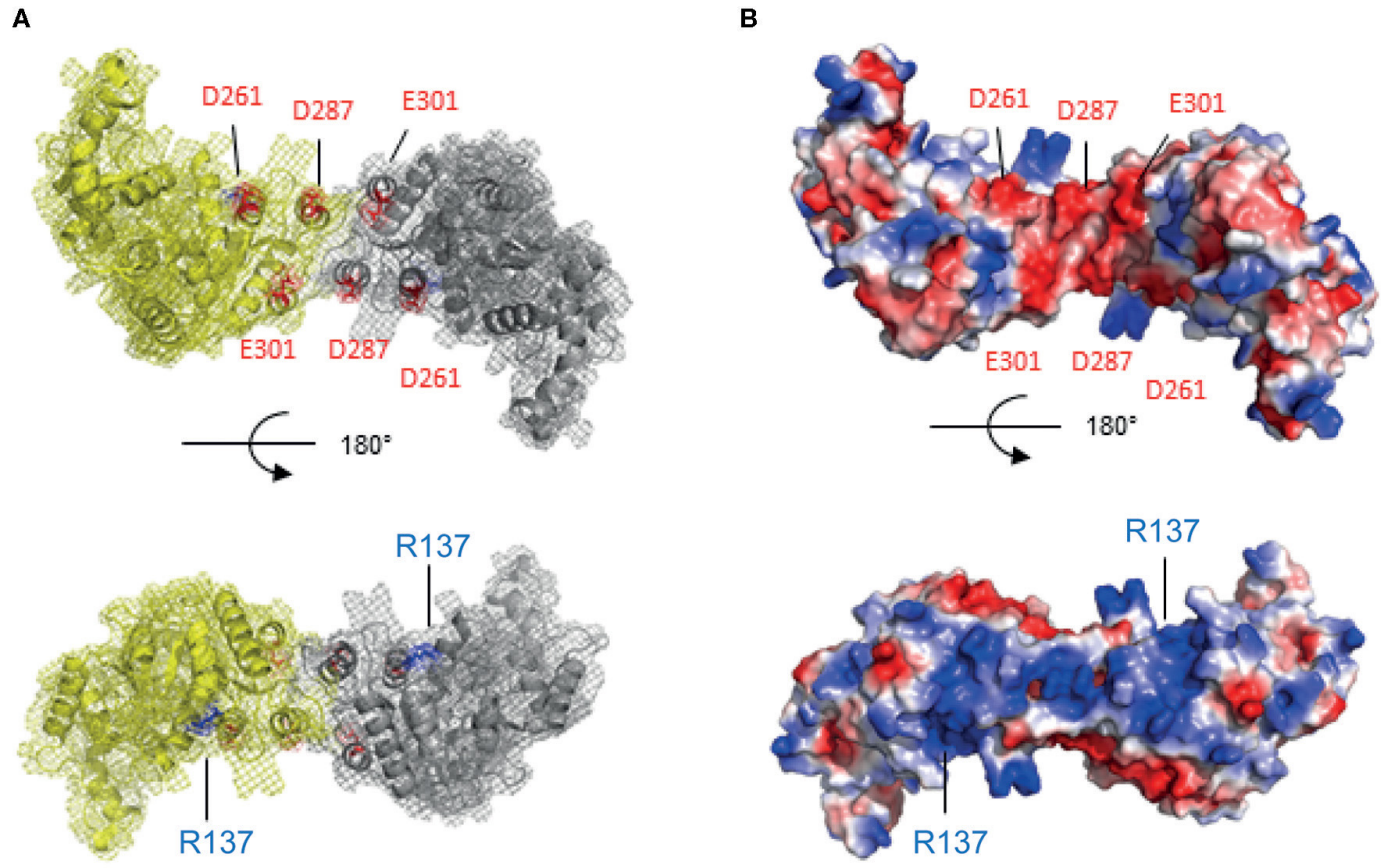
Calculated structural model of DprA dimer from *D. radiodurans*. The WH CTD is missing for clarity. **(A)** Two views as ribbon schematic presentations and surface mesh. **(B)** Same two views of the electrostatic surface of the protein. The knowledge of Sp and Hp DprAs, the electronegative face (in red) in the interaction with RecA via the acidic residues D261, D287, and E301 (top), and the electropositive face (in blue), could thus be involved in the DNA interaction via the R137 residue (bottom).

The recombination mediator DprA protein plays a central role in *B. subtilis* and *S. pneumoniae* transformation by facilitating the loading of RecA protein on single-stranded DNA covered with SSB (Morrison et al., 2007; Quevillon-Cheruel et al., 2012; Lisboa et al., 2014). A putative *D. radiodurans* homolog of the *dprA* gene (*dr0120*) (Table 1) encodes a protein harboring the 3 characteristic domains of the DprA-family: an N-terminal domain [Sterile Alpha Motif (SAM fold)], a central dimeric domain (Rossmann Fold) and a C-terminal domain [Winged Helix (WH fold)] (Figure S3). Using the SWISS MODEL software (Waterhouse et al., 2018), the high sequence identity of 39.47% between DrDprA and *Rhodopseudomonas palustris* DprA (PDB ID: 3MAJ) allowed us to build a convincing 3D structural model (Figure 3A). The model cover is almost complete and highly reliable, with a coverage from residues Ala9 to Arg370 (the end of DrDprA) and a QMEAN of −0.91.

The transformation frequency of cells devoid of DprA by genomic and plasmid DNA dropped 160- and 21- fold, respectively (Figures 2A,B), contrasting with the minor effect of *recO* and *recF* deletions. In the Δ*dprA* mutant, the expression of *dprA* from the ectopic locus *amyE* restored a wild-type level of transformation frequency (Figure S2). These results suggest a major role of DprA in DNA transformation, as reported in other bacteria, probably by facilitating the loading of RecA protein onto ssDNA.

**Figure 4:**
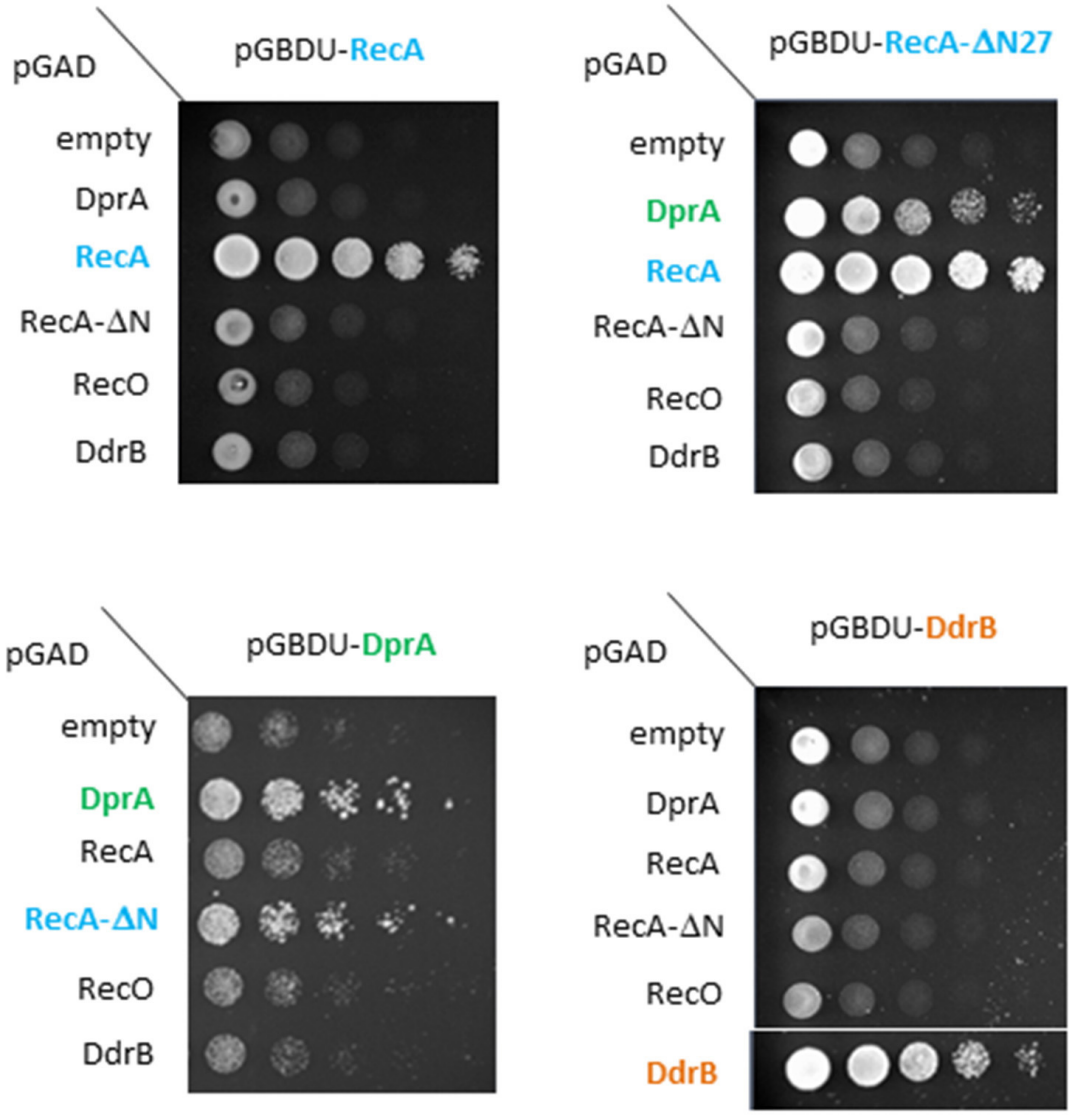
Deinococcal DprA and RecA proteins directly interact. Yeasts expressing DprA, RecA, RecA-ΔN27, or DdrB as Gal4-BD fusions and DprA, RecA, RecA-ΔN27, RecO, or DdrB as Gal4-AD fusions were spotted as a series of 1/5 dilutions on selective medium lacking leucine, uracil, and histidine. Plates were incubated for 5 days at 28°C.

Loading of RecA onto the invading ssDNA requires an interaction between SpDprA and SpRecA in *S. pneumoniae* (Mortier-Barriere et al., 2007). Our 3D structural model of DrDprA harbors the electronegative patch found at the surface of SpDprA involved in the interaction with SpRecA (Figure 3B, top). Therefore, we hypothesized that the interplay between DrDprA and DrRecA might use the same strategy, based on electrostatic interactions between electronegative Asp and Glu residues of DprA and electropositive Arg and Lys residues of RecA. Indeed, we observed, using the yeast two-hybrid system, that DrDprA and DrRecA can efficiently interact in an intracellular context (Figure 4). As for the *S. pneumoniae* system (Lisboa et al., 2014), the first 27 amino acids of RecA, involved in its polymerization, have to be removed to reveal the interaction between DrDprA and DrRecA (Figure 4). Together, these results strongly suggest that DrDprA can function as a recombination mediator through RecA loading onto ssDNA *in vivo*, as described in other bacteria.

However, transformation of a Δ*dprA* mutant by genomic DNA was not completely abolished (Figure 2A). We postulated that the RecFOR pathway could be involved in these residual events. In order to test this hypothesis, we constructed Δ*dprA* Δ*recF* and Δ*dprA* Δ*recO* double mutants. The transformation frequency of genomic DNA was completely abolished in these double mutants (Figure 2A) suggesting that, in the absence of DprA, the RecFOR complex may favor the loading of RecA protein on the internalized ssDNA.

Interestingly, we also observed, in the absence of DprA, a 21-fold reduction of the transformation frequency by plasmid DNA (Figure 2B), even though these transformation events do not involve homologous recombination between plasmid and host genome DNA. This result suggests that DprA has a role in plasmid transformation different from its requirement for RecA loading.

**Figure 5:**
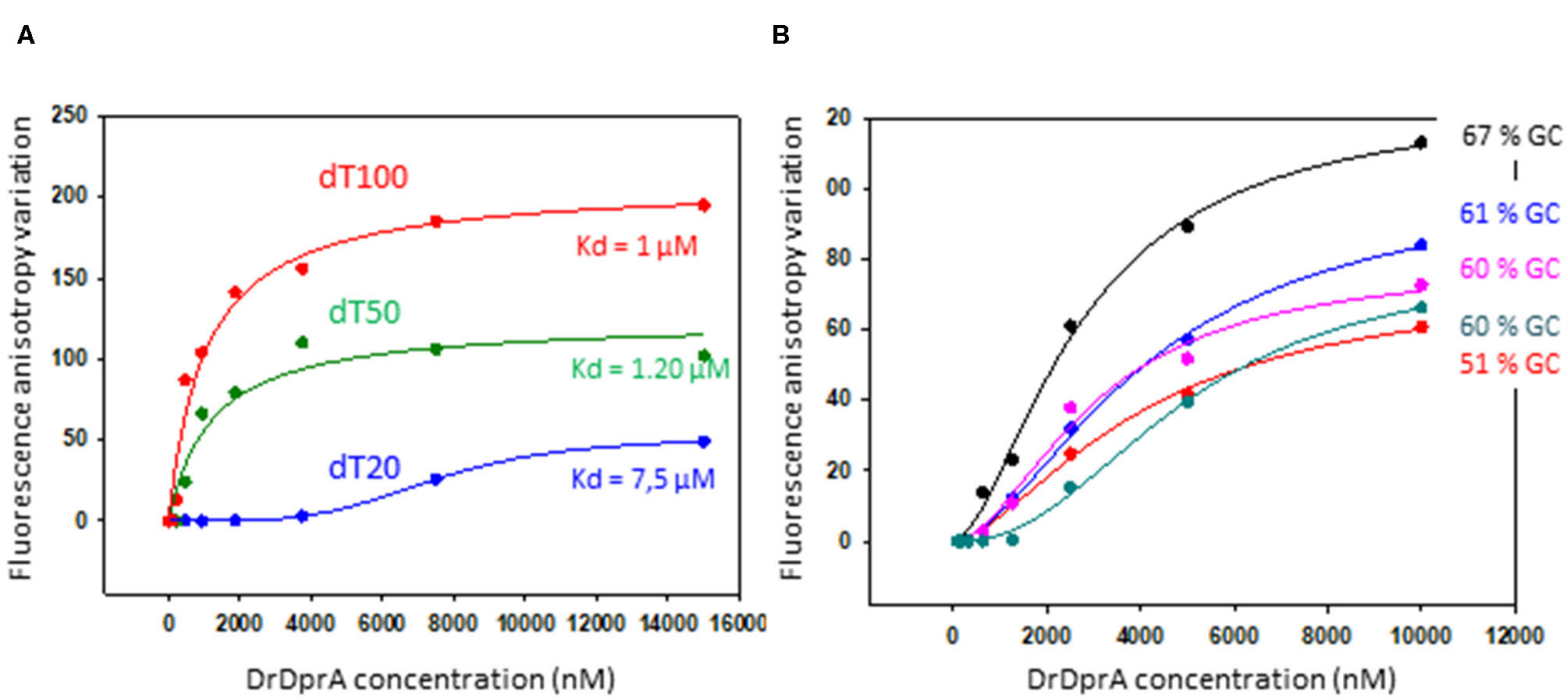
DNA binding analysis of DprA. Equilibrium binding of DprA to fluorescein-labeled dT20, dT50, or dT100 ssDNA **(A)** or 35-mer oligonucleotides of various GC content percentage **(B)**. Fluorescence anisotropy variation with increasing concentrations of DprA fit to a single-ligand–binding model with SigmaPlot-calculated (apparent) K_d_ of 7.5, 1.2, and 1 μM for the three poly-dT, respectively.

DprA from *B. subtilis* and *S. pneumoniae* are involved, in addition to SSB, in the protection of the internalized ssDNA from degradation. Using a purified recombinant DrDprA protein, we first demonstrated its ability to bind ssDNA *in vitro*. The affinity of the protein increased with the length and the GC enrichment of the ssDNA substrate (Figure 5). The affinity for the ssDNA is of the same order of magnitude as the other studied DprAs (Lisboa et al., 2014; Dwivedi et al., 2015). One arginine residue has been shown to be important by biochemical and structural studies in *S. pneumoniae* and *H. pylori* DprA (R115 and R52, respectively). Because this residue is strictly conserved in all known DprA proteins (Figure S3), we unambiguously designated the R137 residue of DrDprA as the major determinant for the interaction of the electropositive face of DprA with ssDNA (Figure 3B, bottom). These results indicate that DrDprA has the same properties as the other studied DprA and might be involved in ssDNA protection when binding to the ssDNA and therefore protect the incoming ssDNA in plasmid transformation as well.

### The N-Terminal SAM Domain of DprA May Be Implicated in the Expression or the Stability of the Protein

As mentioned earlier, DrDprA is a modular protein containing 3 domains, as described in *R. palustris* (Figure S3): a central Rossmann Fold domain, an N-terminal SAM domain and, a C-terminal WH domain (Dwivedi et al., 2015; Lisboa et al., 2019). However, the organization around the central Rossmann Fold domain of DprA from different species is variable, as one of the two C-terminal and N-terminal domains may be absent.

**Figure 6:**
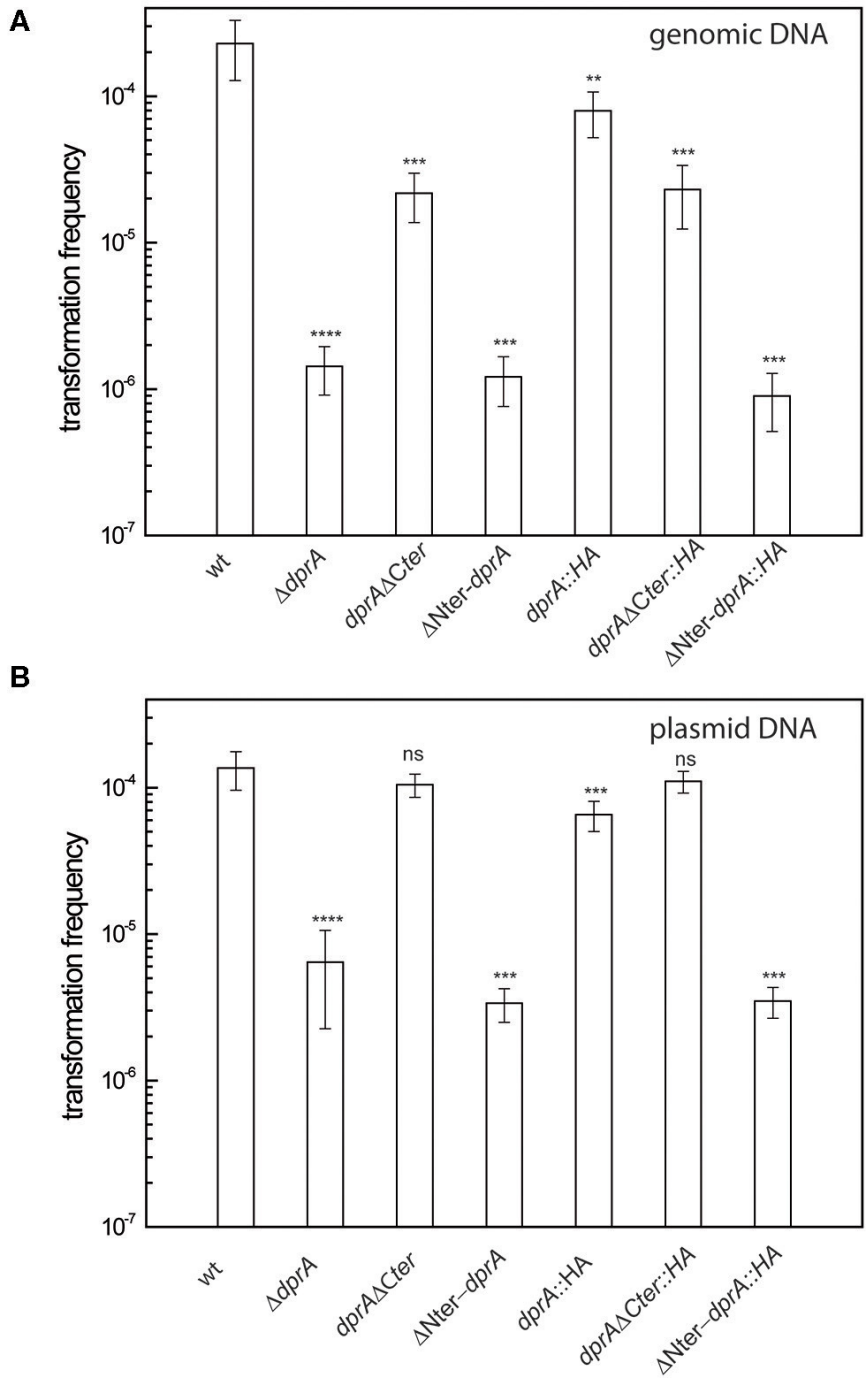
Effect of DprA domain deletions on transformation frequency. *D. radiodurans* cells were transformed with **(A)** 200 ng genomic DNA from a Rif^R^ (GY 11733) strain or **(B)** with 200 ng of p11559 plasmid DNA conferring spectinomycin resistance. The results are the average of at least five independent experiments. Statistically significant differences of transformation frequencies of the mutants, compared with those observed in wild type strain, were calculated using the non-parametric Mann Whitney test: **P < 0.01, ***P < 0.001, ****P < 0.0001; ns if *P* > 0.5.

Thus, we investigated whether the deletion of the N-terminal (Δ80_DprA) or the C-terminal (DprA_Δ63) residues of the *D. radiodurans* DprA protein affected transformation frequency. For this purpose, we constructed *D. radiodurans* mutants expressing the truncated forms of DprA by allelic replacement of the wild type *dprA* gene. The absence of the C-terminal domain only decreased the transformation frequency of genomic DNA 10-fold (Figure 6A) and did not affect plasmid transformation (Figure 6B). On the other hand, in the absence of the DprA N-terminal domain, the transformation frequency of genomic DNA and plasmid DNA (Figure 6) is reduced by 190- and 40-fold, respectively, conferring a phenotype very similar to those of a Δ*dprA* strain (Figure 2).

**Figure 7:**
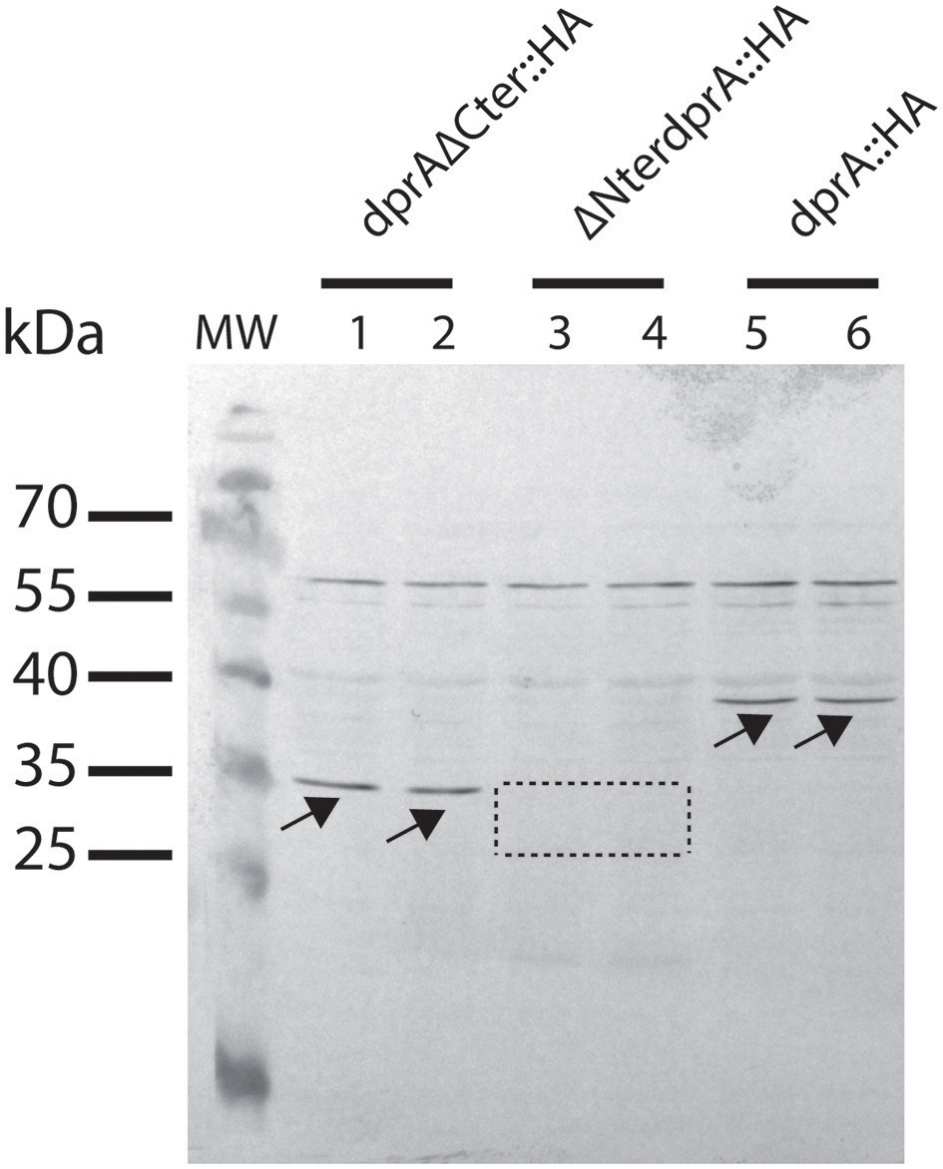
Expression of DprA and truncated DprA proteins in *D. radiodurans*. Strains GY 16054 (*dprA*::HA-*kan*), GY 16201 (ΔNter*dprA*::HA-*kan*), and GY 16058 (*dprA*ΔCter::HA-*kan*) cell extracts were subjected to SDS-PAGE and analyzed by western blotting with anti-HA antibodies. DprA::HA (38 kDa) and DprAΔCter::HA proteins (35 kDa) are indicated with arrows. The area where ΔNterDprA::HA protein (33 kDa) is expected is indicated by a dotted line.

Since the expression and the stability of the truncated proteins can be modified with respect to that of the wild-type protein, strains expressing the intact and the truncated proteins fused to an HA tag were constructed to verify protein expression by Western Blot analysis. As shown in Figure 6, the transformation frequency obtained in the strains expressing the DprA::HA protein and the truncated proteins fused to a HA-tag were the same as those obtained in strains expressing the proteins without the HA-tag (Figure 6), indicating that this tag did not alter the functionality of these proteins. While DprA::HA (41 kDa) and DprAΔCter::HA (35 kDa) were detected by Western Blot in similar amounts, ΔNter DprA::HA protein (33 kDa) was not detected (Figure 7). Therefore, the lower transformation frequency of ΔNter *dprA* mutant strain, which is comparable to that of Δ*dprA* mutant, is likely due to a reduced stability of this truncated protein or a lower expression of the ΔNter *dprA* gene.

Taken together, these results suggest that the C-terminal domain of DrDprA is not essential for DNA protection of the internalized DNA, but rather plays a role in the interaction with the RecA protein and the loading of RecA on the internalized genomic ssDNA, whereas the N-terminal domain is required for the correct folding of the protein or for its stability.

### DprA Is Not Implicated in the Radioresistance of *D. radiodurans*

**Figure 8:**
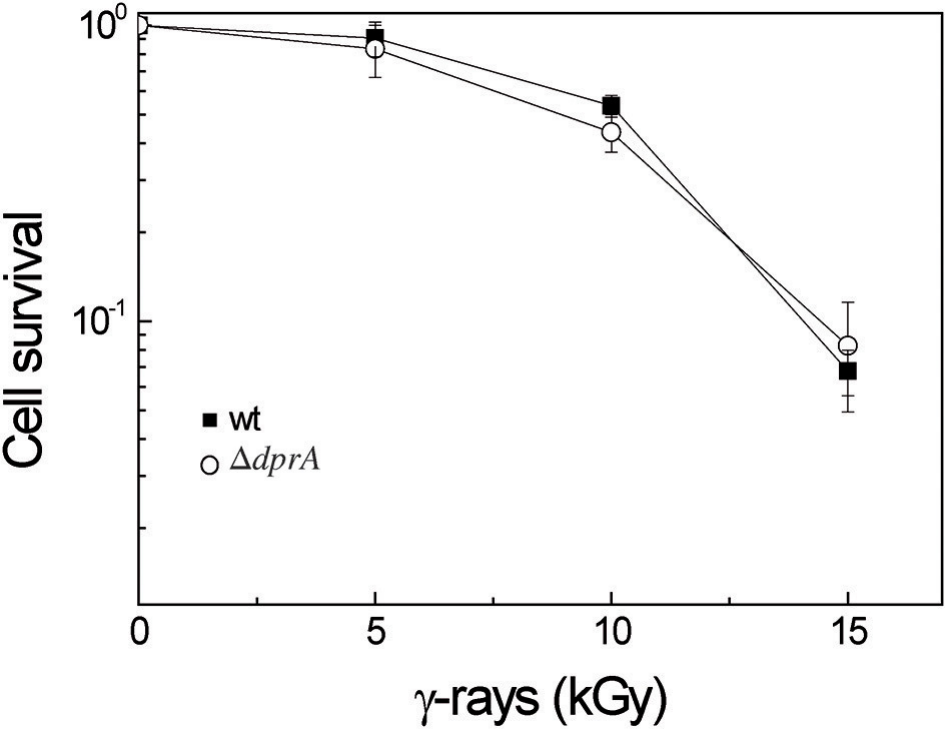
DprA is not involved in *D. radiodurans* radioresistance. Bacteria were exposed to γ-irradiation at doses indicated on the abscissa. Wild type (closed squares), Δ*dprA* (open circles).

Because of its ability to protect ssDNA against degradation and its interaction with the RecA protein, we also investigated if DprA might participate in *D. radiodurans* radioresistance. Survival after exposure to γ-irradiation, at doses ranging from 0 to 15 kGy, was measured (Figure 8). The Δ*dprA* mutant showed the same resistance to γ-radiation as the wild type strain, suggesting that DprA was not required to protect ssDNA generated after irradiation during maturation of the DNA double strand breaks and in the ESDSA repair pathway. The interaction of DprA with the RecA protein is not crucial for ESDSA or recombinational repair of the DNA double strand breaks, confirming the major role of the RecFOR proteins in these repair pathways (Bentchikou et al., 2010).

### DdrB Is Required for Transformation of Cells Devoid of DprA

DdrB, a protein specific to *Deinococcaceae*, binds to ssDNA and exhibits some biochemical properties similar to those of the *E. coli* SSB protein (Norais et al., 2009). Whereas the absence of DdrB had no influence on the transformation efficiency of genomic DNA, it strongly affected the frequency of transformation by plasmid DNA (Bouthier de la Tour et al., 2011). We have previously proposed that DdrB likely participates to the plasmid establishment through its single strand annealing activity (Bouthier de la Tour et al., 2011). Here, we obtained the same result (Figure 2) and showed that expression *in trans* of the *ddrB* gene in a Δ*ddrB* mutant restored a wild-type level of transformation frequency with plasmid DNA (Figure S2B).

However, plasmid transformation is not fully abolished in a Δ*ddrB* mutant, suggesting that other proteins may be able to compensate for its role in plasmid transformation. The frequency of plasmid transformation was completely abolished in Δ*ddrB* Δ*recO and* Δ*ddrB* Δ*recF* double mutants (Figure 2B). These results are difficult to interpret if we take into account the lethal sectoring of the Δ*ddrB* Δ*recO* (Ithurbide et al., 2015) and Δ*ddrB* Δ*recF* double mutants and the reduced efficiency of plasmid transformation when cells are devoid of the DdrB protein. In contrast, the frequencies of genomic transformation of Δ*ddrB*, Δ*ddrB* Δ*recO, and* Δ*ddrB* Δ*recF* bacteria were the same as those measured in wild type bacteria (Figure 2A).

As the ssDNA enters the cell, this becomes a substrate for several single stranded DNA binding proteins, either SSB, DdrB, DprA, RecO, and RecA and thus they all compete at one point with one or several of the others for binding to the ssDNA. Thus, we tested whether some of these proteins could interact among each other using the yeast two-hybrid system. As mentioned earlier, DprA and RecA are able to form homodimers and this interaction was used as a positive control for interaction in these studies (Figure 4). No interaction was detected between DdrB/DprA, DprA/RecO nor DdrB/RecO (Figure 4).

Interestingly, we also showed that transformation by plasmid DNA, and also by genomic DNA, was completely abolished in a Δ*dprA* Δ*ddrB* double mutant (Figure 2), suggesting that, in the absence of DprA, DdrB might participate in the protection of internalized ssDNA during transformation.

## Discussion

**Figure 9:**
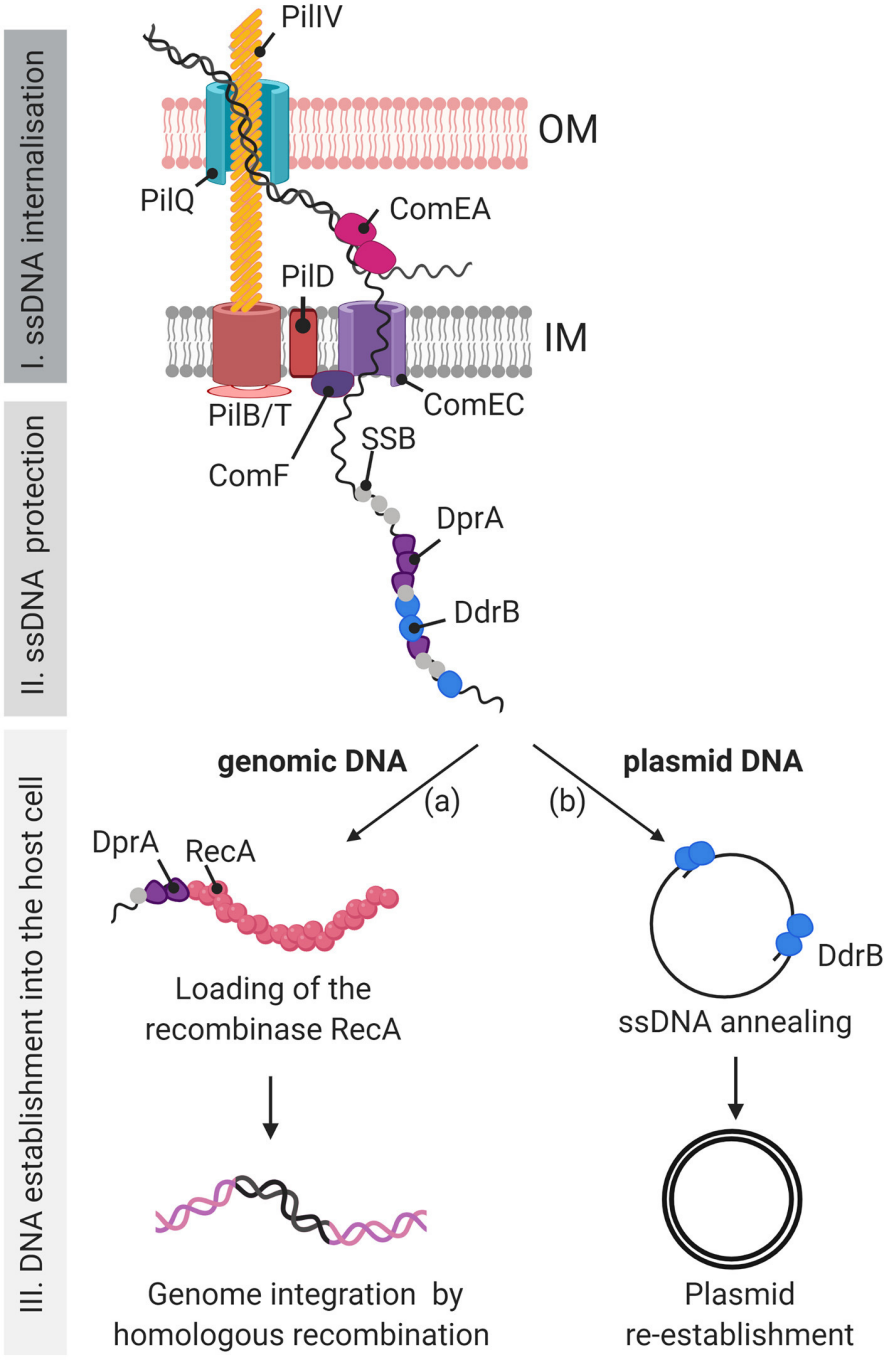
Model of the DNA uptake apparatus and DNA processing during natural transformation in *D. radiodurans*. Our study suggests that, during natural transformation, the exogenous DNA is internalized into the *D. radiodurans* cytosol via a type IV-like DNA uptake machinery, similar to that of *V. cholerae*, which is composed of homologues of the pilins PilIV (DR0548 and DR1232), the outer membrane (OM) channel PilQ (DR0774), the ATPases PilB (DR1964) and PilT (DR1963), and the pre-pilin peptidase PilD (DR2065). We present here a hypothetical inner membrane channel which could be similar to PilC in *V. cholerae* (Matthey and Blokesch, 2016). (I) The external DNA passes through the OM via the PilQ channel and the DNA binding protein ComEA (DR1855) pulls the DNA into the periplasm. Only one DNA strand is translocated into the cytoplasm through the ComEC (DR1854) inner membrane (IM) channel, probably assisted by ComF (DR1389). (II) The ssDNA is protected after its entry in the cell by ssDNA binding proteins such as SSB, DdrB and DprA (DR0120) (III) Plasmid and genomic DNA are differentially processed. (IIIa) DprA, bound to genomic DNA, will favor the loading of RecA onto the genomic DNA to initiate homologous recombination with the genome of the host cell. (IIIb) In a RecA independent manner, DdrB, via its single strand annealing (SSA) activity, favors the annealing of plasmid DNA fragments to allow DNA replication, reconstitution of a circular double-stranded DNA molecule, and establishment of the plasmid in the host cell. The RecO protein is proposed to play a major role as a back-up transformation protein for both the genomic and plasmid DNA processing pathways either by replacing DdrB for the annealing of the plasmid DNA fragments, or by replacing DprA for the loading of RecA onto genomic DNA prior to recombination through the RecFOR pathway (not presented here).

*D. radiodurans* is the object of major interest due to its exceptional capacity to resist high levels of radiation and to reconstruct a functional genome from hundreds of radiation-induced chromosomal fragments. Homologous recombination is a mechanism for repairing DNA double-strand breaks and contributes to preserve the integrity of genomes. It also plays an important role in horizontal gene transfer and, more particularly, in bacterial transformation and thus in the evolution of genomes. Although *D. radiodurans* natural transformation allowed it's use as a major model organism to study radioresistance, little is known about the proteins and mechanisms involved in its transformation. Here, we investigate the involvement of different Deinococcal proteins in natural transformation and propose a model showing their role at the three stages of the process: DNA internalization, DNA protection and DNA establishment into the host cell (Figure 9).

Initial stages of DNA transformation involve the internalization of external DNA into the cytosol and therefore the passage of the DNA through the different layers of the cell envelope, a step that represents different challenges in monoderm or diderm bacteria. Although *D. radiodurans* stains Gram-positive in a manner similar to monoderm bacteria, *D. radiodurans* possesses an outer membrane (Thompson and Murray, 1981). However, *D. radiodurans* lacks the lipopolysaccharides (LPS) considered as a defining characteristic of the Gram-negative group (Gupta, 2011). Due to this cell envelope characteristic, *D. radiodurans*, and more generally the *Deinococcus-Thermus* phylum, is proposed to be an intermediate lineage in the monoderm-diderm transition in bacterial evolution and that the LPS appeared later in evolution (Gupta, 2011). Due to this difference in cell envelope structure, Gram-negative and Gram-positive bacteria have different DNA uptake apparatus (Mell and Redfield, 2014). Where most of the competent species from both groups share the presence of ComEA (Seitz et al., 2014), a DNA receptor, and ComEC, a conserved membrane pore mediating the transfer of one DNA strand through the inner membrane (Pimentel and Zhang, 2018), Gram-negative bacteria possess another pore located in the outer membrane. This pore is formed by PilQ (Seitz et al., 2014) and the external DNA is pulled through by a type IV derived competence pilus. Here, after a genomic survey for putative DNA uptake genes, we report for the first time the major proteins involved in the competence pseudo-pilus and the DNA translocation machinery in *D. radiodurans*. We showed that the DNA translocating machinery of *D. radiodurans* is related to type IV pili with the predicted PilQ (DR0774) protein, the pilins DR0548 and DR1232, the PilD (DR2065) peptidase subunit and the PilB (DR1964), PilT (DR1963) ATPases being essential for transformation. In addition, *D. radiodurans* homologs of ComEA (DR1855) and ComEC (DR1854), generally found in competent bacteria, are similarly essential for transformation. The presence in the cells of a second homolog of ComEA (DR0207) and ComEC (DR0361) was not sufficient to compensate the absence of DR1855 and DR1854. The results obtained in this study suggest that the model of the DNA-uptake machinery in *D. radiodurans* is similar to that extensively described in the Gram-negative bacterium *V. cholerae* (Seitz and Blokesch, 2013b) (Figure 9, I). Further and in depth studies of the DNA uptake apparatus in *D. radiodurans* could potentially provide insights into the evolution of the Type 4 apparatus in relation to the evolution of the outer layers and competence in the transition from monoderm to diderm bacteria.

In bacteria, competence development is often tightly regulated in response to specific environmental signals and is usually limited to a small time-frame window during the growth cycle [for review, see (Seitz and Blokesch, 2013a)]. Different signals, such as genotoxic stresses causing DNA damage, including UV light or some antibiotic treatments (Dorer et al., 2011; Corbinais et al., 2016), quorum sensing (Havarstein et al., 1996; Claverys et al., 2006; Boudes et al., 2014), or starvation for carbon sources (Redfield, 1991) are needed to trigger the physiological state of competence. Competence regulatory circuits are not universal and differ according to the species. In *B. subtilis*, the transcriptional regulator ComA regulates the development of genetic competence (Comella and Grossman, 2005) and is part of, along with ComP, a two component system. An analog of ComP was not described in *D. radiodurans*. *D. radiodurans*, as are *Neisseria gonorrhoeae* and *H. pylori*, is constitutively competent throughout it's growth phases (Tirgari and Moseley, 1980; Aas et al., 2002; Dorer et al., 2011). In *D. radiodurans*, in the absence of the DR0847 protein, annotated as being a member of the ComA family, the transformation frequency is the same as the wild type strain, suggesting that DR0847 is not involved in the regulation of genetic competence in this bacterium.

We also studied the implication of several recombination and DNA repair proteins during natural transformation of *D. radiodurans*. During internalization, the exogenous ssDNA is taken in by numerous proteins allowing its protection from nuclease attack and integration into the genome (Figure 9, II and III). These steps mainly involve single-strand DNA binding proteins, homologous recombination mediator proteins and RecA recombinase in the different bacterial species studied so far [for review (Kidane et al., 2012; Johnston et al., 2014)]. The transformation frequency of genomic DNA that requires integration of the DNA into the genome is completely abolished in cells devoid of the RecA protein. In *S. pneumoniae*, the incoming DNA is immediately degraded in the absence of RecA, demonstrating that the internalized ssDNA requires protection prior to the search for homology and that RecA is needed for this protection (Berge et al., 2003). The reduction of plasmid DNA transformation frequency in the absence of the RecA protein in *D. radiodurans* suggests that, as in *S. pneumoniae*, RecA has a role in protecting incoming ssDNA during the first step of the natural transformation process, corresponding to DNA internalization, although it might not be the key player, as the reduction is not drastic. In *S. pneumoniae* and *B. subtilis*, RadA is involved in the transformation by genomic DNA (Kruger et al., 1997; Carrasco et al., 2002; Burghout et al., 2007). Here, we showed that RadA is not involved in *D. radiodurans* transformation.

Numerous studies about homologous recombination in DNA repair have shown that RecA requires Recombination Mediator Protein (RMP) for its loading onto single stranded DNA. In *D. radiodurans*, we observed a 160-fold reduction of genomic DNA transformation in the absence of DrDprA. Therefore, we propose that, as previously demonstrated in *S. pneumoniae* (Quevillon-Cheruel et al., 2012), DrDprA facilitates the loading of RecA protein on ssDNA (Figure 9, IIIa), a hypothesis supported by the positive results of the yeast two-hybrid test between DrDprA and DrRecA. Interestingly, genomic transformation is not completely abolished in the absence of DprA. Although deletion of *recO* or *recR*, encoding proteins of the RecFOR RMP complex, had only a slight effect on genomic DNA transformation efficiency, genomic DNA transformation was completely abolished in Δ*dprA* Δ*recF* and Δ*dprA* Δ*recO* double mutants. We propose that the RecFOR complex could partially compensate for the role of DprA as a recombination mediator protein to load RecA on the internalized ssDNA or, alternatively, the RecFOR complex and the DprA protein act at a different step of the recombination process, i.e., they are epistatic.

Although plasmid transformation does not require genome integration of the incoming DNA by homologous recombination, we observed in *D. radiodurans* a 21-fold reduction of plasmid transformation frequency in the absence of the DprA protein. This suggests that DprA may also be involved in the protection of the internalized plasmid DNA fragments by coating the ssDNA, as shown in *S. pneumoniae* (Mortier-Barriere et al., 2007; Quevillon-Cheruel et al., 2012). By extension, we propose that DrDprA might also protect the incoming genomic DNA in addition to favoring the loading of RecA on ssDNA (Figure 9, II). Moreover, Yadav et al. (2013) have shown that *B. subtilis* DprA anneals complementary strands coated by SsbB to reconstitute a dsDNA circular plasmid molecule (Yadav et al., 2013), another role that can be played by DrDprA in plasmid transformation.

In *B. subtilis*, RecO plays a major role during plasmid transformation, whereas it plays only a minor role during chromosomal transformation (Yadav et al., 2013). It was shown that RecO anneals complementary ssDNA complexed with SsbA proteins (Kidane et al., 2009). This activity is required to reconstitute an intact plasmid from ssDNA fragments. In *D. radiodurans*, the RecO protein seems to play only a minor role in plasmid transformation when DdrB is present in the cells, consistent with the low *in vitro* DNA single strand pairing activity of the Deinococcal RecO protein (Makharashvili et al., 2004) and the single strand annealing activity of the DdrB protein (Xu et al., 2010). In contrast, RecF plays a minor role in *B. subtilis* as well as in *D. radiodurans* for plasmid and chromosomal transformation [(Kidane et al., 2009), this work]. The Δ*recO* and Δ*recF* single mutants showed lethal sectoring, and this phenotype is increased in Δ*recO* Δ*ddrB* and Δ*recF* Δ*ddrB* double mutants (Ithurbide et al., 2015). The limit of detection of transformants by plasmid DNA is therefore reached and makes the interpretation difficult. Previously, we have shown that the *Deinococcus* specific protein, DdrB, is involved in a large number of cellular processes: (i) DdrB participates in the genetic instability of repeated sequences via SSA (Ithurbide et al., 2015), (ii) it is probably required to support blocked replication forks if recombination proteins are absent (Ithurbide et al., 2015), (iii) it participates in early genome repair by SSA after exposure to high doses of ionizing radiation (Xu et al., 2010; Bouthier de la Tour et al., 2011), (iv) it favors reconstitution by single strand annealing of plasmid DNA (Bouthier de la Tour et al., 2011) during plasmid transformation (Figure 9, IIIb).

Whereas the frequency of plasmid transformation of the single Δ*ddrB* mutant by plasmid DNA was reduced (Bouthier de la Tour et al., 2011), transformation was completely abolished in a Δ*dprA* Δ*ddrB* double mutant. We also showed that genomic transformation was completely abolished in cells devoid of DprA if the DdrB protein was not present. In the absence of DprA, DdrB might be required during transformation for the protection of internalized ssDNA, a role that might not be played alone by the essential *Deinococcus* SSB protein (DR0100). Thus, in the absence of DprA, DdrB might be involved at two levels in the transformation by plasmid DNA: (i) protection of the ssDNA against degradation due to its SSB-like properties (Figure 9, II) (ii) reconstitution of the plasmid by its SSA activity (Figure 9, IIIb) (Xu et al., 2010; Bouthier de la Tour et al., 2011). Therefore, the DdrB protein, whose expression is strongly induced after irradiation, exhibits pleiotropic roles in the cell that go beyond its role in DNA repair (Tanaka et al., 2004; Bouthier de la Tour et al., 2011).

As for SsbB from *S. pneumoniae*, DdrB loaded on the transforming ssDNA might be a barrier for the recruitment of RecA, requiring the action of a recombination mediator protein. In the absence of DprA, we propose that the RecFOR complex plays this role and alleviates the DdrB barrier to facilitate the loading of RecA by displacing the DdrB protein, as supported by (i) the complete abolition of transformation in absence of both DprA and RecO and (ii) the restoration of the wild type frequency of transformation in the Δ*recO* Δ*ddrB and* Δ*recF* Δ*ddrB* double mutants.

Here, we also demonstrated that DprA is not involved in *D. radiodurans* radioresistance, suggesting that the DprA protein is not required for the loading of RecA or ssDNA protection during recombinational repair and ESDSA repair of DNA double strand breaks, RecFOR being the RMP essential in these processes (Bentchikou et al., 2010). Thus, a high transformation frequency observed in *D. radiodurans* is not required for efficient DNA repair of DNA double strand breaks after irradiation.

In conclusion, as in other naturally competent bacteria, the DprA protein plays a major role in *D. radiodurans* natural transformation, whereas the RecFOR complex and the Deinococcal-specific protein DdrB might partially compensate for the absence of DprA, possibly by favoring the loading of RecA on internalized ssDNA and by protecting it from nuclease attack, respectively.

## Materials and Methods

### Bacterial Strains, Plasmids, Oligonucleotides, Media

Bacterial strains and plasmids are listed in Tables 2, 3, respectively. The *E. coli* strain DH5α was used as the general cloning host and strain SCS110 was used to propagate plasmids prior to introduction into *D. radiodurans* via transformation (Meima et al., 2001). All *D. radiodurans* strains were derivatives of the wild-type strain R1 ATCC 13939. The deletion mutants were constructed by the tripartite ligation method. An antibiotic cassette (kanamycin or chloramphenicol resistance gene) and two 500 bp genomic fragments from upstream and downstream of the coding region of target gene were amplified by PCR using primer pairs that introduced *BamHI* and *XbaI* restriction sites (to construct *pilT* and *pilB* mutant, we used *Xho*I and *Xba*I restriction sites). The three fragments were ligated together in molecular ratio 1/1/1 (100 ng of 500 bp fragments) so that the antibiotic cassette was flanked by the two genomic fragments. The constructs were then introduced into *D. radiodurans* by genetic transformation selecting for antibiotic resistance. This led to the replacement of the wild-type allele by the mutant counterpart via homologous recombination. *D. radiodurans* is multigenomic, with cells containing from 4 to 10 genome equivalents. Homogenotes of the deletion allele were obtained after two or three cycles of purification on selective medium. The genetic structure and purity of the mutants were verified by PCR.

**Table 2:** Bacterial strains used in this study.

**Strains**	**Description**	**Source or reference**
***E. coli***		
*DH5α*	*supE44ΔlacU*(*Φ80lacZΔM15*) *hsdR17 recA1 endA1 gyrA96 thi-1 relA1*	Laboratory stock
*SCS110*	*endA dam dcm supE44 Δ(lac-proAB) (F'traD36 proAB lacI^*q*^ZΔM15)*	Laboratory stock
*BL21(DE3)Gold*	*F^−^ ompT hsdS(rB^−^ mB^−^) dcm^+^Tet^*r*^galλ(DE3) endA Hte*	Laboratory stock
***D. radiodurans***		
R1/GY9613	ATCC 13939	Laboratory stock
GY 11733	*rif*Δ9	Mennecier et al., 2004
GY 12835	Δ*ddrB*Ω*kan*	Bouthier de la Tour et al., 2011
GY 12958	Δ*radA*Ω*cat*	Ithurbide et al., 2015
GY 12965	Δ*recF*Ω*cat*	Bentchikou et al., 2010
GY 12966	Δ*recO*Ω*hph*	Bentchikou et al., 2010
GY 12968	Δ*recA*Ω*kan*	Bentchikou et al., 2010
GY 12970	Δ*radA*Ω*cat* Δ*recA*Ω*kan*	GY12958 × GY12968 DNA
GY 13915	Δ*ddrB*Ω*cat*	Ithurbide et al., 2015
GY 15121	Δ*dprA*Ω*kan*	This work^a^
GY 16052	Δ*recO*Ω*cat*	This work^a^
GY 16054	*dprA*::HAΩ*kan*	This work^a^
GY 16056	ΔNter *dprA*Ωcat	This work^a^
GY 16058	*dprAΔCter*Ω*cat*	This work^a^
GY 16084	Δ*dprA*Ω*kan* Δ*recF*Ω*cat*	GY12965 x GY15121 DNA
GY 16086	Δ*dprA*Ω*kan* Δ*recO*Ω*cat*	GY16052 x GY15121 DNA
GY 16683	Δ*dr1854*-*dr1855*Ω(*comEC*/*comEA*)Ω*kan*	This work^a^
GY16201	ΔNter *dprA::HA*Ω*kan*	This work^a^
GY 16203	Δ*ddrB*Ω*cat* Δ*dprA*Ω*kan*	GY13915 x GY15121 DNA
GY 16205	*dprAΔCter::HA*Ω*kan*	This work^a^
GY 16956	Δ*recF*Ω*cat* Δ*ddrB*Ω*kan*	GY12965 X GY12835 DNA
GY 16964	Δ*amyE*::P*_*dr*1963_*_*dr1963*Ω*kan* Δ*dr1963*Ω*cat*	This work^a^
GY 16966	Δ*amyE*::P*_*dr*1963_*_*dr1963*Ω*kan* Δ*dr1963*Ω*cat*	GY16964 X GY17790 DNA
GY 17018	Δ*dr0774(pilQ)*Ω*cat*	This work^a^
GY 17019	Δ*dr0207(comEA)*Ω*cat*	This work^a^
GY 17020	Δ*dr2065(pilD)*Ω*cat*	This work^a^
GY 17021	Δ*fimA*Ω*cat*	This work^a^
GY 17052	Δ*ddrB*Ω*cat* Δ*recO*Ω*hph*	GY13915 x GY12966 DNA
GY 17130	Δ*amyE*Ω*cat*	This work^a^
GY 17132	Δ*amyE*::P*_*dr*0548_*_*dr0548*Ω*cat*	This work^a^
GY 17134	Δ*amyE*::P*_*dr*1854_*_*dr1854*-*dr1855*Ω*cat*	This work^a^
GY 17136	Δ*amyE*::P*_*dprA*_*_*dprA*Ω*cat*	This work^a^
GY 17138	Δ*amyE*::P*_*dr*1389_*_*dr1389*Ω*cat*	This work^a^
GY 17140	Δ*amyE*Ω*kan*	This work^a^
GY 17144	Δ*amyE*::P*_*dr*2065_*_*dr2065*Ω*kan*	This work^a^
GY 17148	Δ*amyE*::P*_*recO*_*_*recO*Ω*kan*	This work^a^
GY 17152	Δ*amyE*::P*_*dr*1964_*_*dr1964*Ω*kan*	This work^a^
GY 17154	Δ*amyE*::P*_*spac*_*_*recA*Ω*cat*	This work^a^
GY 17156	Δ*amyE*::P*_*dr*0548_*_*dr0548*Ω*cat* Δ*dr0548*Ω*kan*	GY17133 X GY17784 DNA
GY 17158	Δ*amyE*::P*_*dr*1389_*_*dr1389*Ω*cat* Δ*dr1389*Ω*kan*	GY17138 X GY17788 DNA
GY 17160	Δ*amyE*::P*_*dr*2065_*_*dr2065*Ω*kan* Δ*dr2065*Ω*cat*	GY17144 X GY17020 DNA
GY 17162	Δ*amyE*::P*_*recO*_*_*recO*Ω*kan* Δ*recO*Ω*cat*	GY17148 X GY16052 DNA
GY 17166	Δ*amyE*::P*_*dr*1854_*_*dr1854-1855*Ω*cat* Δ*dr1854-dr1855*Ω*kan*	GY17134 X GY16683 DNA
GY 17169	Δ*amyE*::P*_*dprA*_*_*dprA*Ω*cat* Δ*dprA*Ω*kan*	GY17136 X GY15121 DNA
GY 17170	Δ*amyE*::P*_*spac*_*_*recA*Ω*cat* Δ*recA*Ω*kan*	GY17154 X GY12968 DNA
GY 17175	Δ*amyE*::P*_*dr*1964_*_*dr1964*Ω*kan* Δ*dr1964*Ω*cat*	GY17152 X GY17792 DNA
GY 18223	Δ*amyE*::P*_*ddrB*_*_*ddrB*Ω*kan* Δ*ddrB*Ω*cat*	GY18099 X GY13915 DNA
GY 17782	Δ*dr1232(pilIV)*Ω*cat*	This work^a^
GY 17784	Δ*dr0548(pilIV)*Ω*kan*	This work^a^
GY 17786	Δ*dr0847(comA)*Ω*cat*	This work^a^
GY 17788	Δ*dr1389(comF)*Ω*kan*	This work^a^
GY 17790	Δ*dr1963*(*pilT*)Ω*cat*	This work^a^
GY 17792	Δ*dr1964*(*pilB*)Ω*cat*	This work^a^
GY 17796	Δ*dr0361(comEC)*Ω*kan*	This work^a^
GY 18091	Δ*amyE*::P*_*recF*_*_*recF*Ω*kan*	This work^a^
GY 18097	Δ*amyE*::P*_*recF*_*_*recF*Ω*kan* Δ*recF*Ω*cat*	GY18091 X GY12965 DNA
GY 18099	Δ*amyE*::P*_*ddrB*_*_*ddrB*Ω*kan*	This work^a^

a *strains were constructed by the tripartite ligation method*.

**Table 3:** Plasmid used in this study.

**Plasmids**	**Description**	**Source or reference**
pPS6	Source of chloramphenicol cassette	Passot et al., 2015
p11086	Source of kanamycin cassette	Laboratory stock
p11559	Shuttle vector *E. coli*/*D. radiodurans*, Spec^R^	Laboratory stock
p11562	p11559 with a fragment encoding RecA, P*_*Spac*_*::*recA*	Jolivet et al., 2006
P12764	Source of HA-tag kanamycine cassette	Toueille et al., 2012
pET29	Expression vector	Novagen

We used the same strategy (tripartite ligation procedure) to construct strains expressing ectopically (in an *amyE* locus) genes to perform complementation assays in the corresponding deletion background. The gene of interest (promoter + coding sequence), a 550 bp genomic fragment located upstream *amyE*, and a fragment containing an antibiotic cassette and the downstream region of *amyE* (using genomic DNA of GY 17130 or GY 17140 strains as template) were amplified by PCR using primer pairs that introduced restriction sites. After enzymatic digestion, the three fragments were ligated and the ligation mixture was used to transform the wild type *D. radiodurans* strain. The genetic structure and purity of the mutants were verified by PCR and sequencing. These strains were transformed with genomic DNA of a strain containing a deletion of the corresponding gene to obtain the complemented strains (see Table 2). The *recA* gene, which is the third gene of an operon, is expressed from the P*spac* promoter and was amplified using p11562 plasmid DNA as template.

The sequence of oligonucleotides used for strain construction and diagnostic PCR are listed in Table S2. Chromosomal DNA of *D. radiodurans* was extracted as previously described (Norais et al., 2013). PCR amplification of DNA fragments, using plasmid or genomic DNA as a template, was performed using Phusion DNA polymerase (Thermo Scientific) or GoTaq Flexi G2 (Promega).

*D. radiodurans* strains were grown at 30°C in TGY2X (1% tryptone, 0.2% dextrose, 0.6% yeast extract), or plated on TGY1X containing 1,5% agar, and *E. coli* strains were grown at 37°C in LB (Lysogeny Broth). When necessary, media were supplemented with the appropriate antibiotics used at the following final concentrations: kanamycin, 6 μg/mL; chloramphenicol, 3 μg/mL; rifampicin, 25 μg/mL; spectinomycin, 80 μg/mL, hygromycin 50 μg/mL for *D. radiodurans*, and 40 μg/mL for *E. coli*.

### Transformation of *D. radiodurans*

To prepare naturally competent cells, exponentially growing bacteria were harvested by centrifugation, re-suspended at a concentration of 5 × 10^8^ cells/mL in TGY2X medium supplemented with 30 mM CaCl_2_ and 10% (V/V) glycerol, and immediately stored at −80°C. For transformation, aliquots (100 μL) of competent cells were thawed on ice and mixed with an equal volume of TGY containing 30 mM CaCl_2_ before DNA (genomic or plasmid DNA) was added. After 20 min at 0°C and 60 min at 30°C, 800 μL of TGY2X were added and the cells were incubated for a further 5 h to allow expression of rifampicin (transformation by genomic DNA) or spectinomycin (transformation by plasmid DNA) resistance. Diluted samples were plated on TGY plates containing the appropriate antibiotics.

### γ-Irradiation Assay

To measure cell survival after exposure to ionizing radiation, exponential phase cultures, grown in TGY2X, were concentrated to an A_650nm_ = 20 in TGY2X media and irradiated on ice with a ^60^Co-irradiation system (Centre d'Energie Atomique, Saclay, France) at a dose rate of 6,000 Gy/h. Following irradiation, diluted samples were plated on TGY plates. Colonies were counted after 3–4 days incubation at 30°C.

### Western Blot Analysis

Mutant *dprA*::HAΩ*kan*, ΔNter-*dprA*::HAΩ*kan* and *dprA*ΔCter::HAΩ*kan* bacteria were grown to an A_650nm_ of 0.4–0.6 in TGY2X and 20 mL of culture was centrifuged. The cell pellets were resuspended in 150 μL of SSC 1X buffer and the cells disrupted as described previously (Bouthier de la Tour et al., 2009). After centrifugation, the protein concentration was measured (using the Bio-Rad protein assay dye reagent kit), and 10 μg of proteins were subjected to electrophoresis through a 12% Glycine SDS polyacrylamide gel. The proteins were transferred onto a PVDF (polyvinylidene difluoride) membrane. The membrane was blocked with TBS containing 5% powdered milk and 0.05% Tween 20 before being incubated overnight at 4°C with a 1:5000 dilution of polyclonal rabbit anti-HA antibodies (Life Technologies) in TBS containing 3% powdered milk, 0.05% Tween 20. After extensive washes in TBS-0.05% Tween 20, the membrane was incubated with anti-rabbit IgG alkaline phosphatase conjugate (Promega) used as secondary antibody and revealed by a colorimetric reaction.

### DprA Overexpression and Purification

Cloning of the *dprA* coding region was performed using genomic DNA from wild type *D. radiodurans* as a template for PCR. Six histidine codons were added at the 3' end of the construct during the PCR process. The fragments were inserted into the *Nde*I-*Xho*I sites of the pET29 vector (Novagen). Over-expression of the protein in the BL21(DE3) Gold strain was performed in 800 ml 2xYT erlenmayer flasks, overnight at 15°C, after induction with 0.5 mM IPTG (Sigma). Cells were harvested by centrifugation and resuspended in buffer A [200 mM NaCl, 20 mM Tris-HCl (pH 7.5)] for all of the constructs. The cells were stored overnight at −20°C. Cell lysates were prepared by sonication using a Branson probe-tip sonicator. After 30 min centrifugation at 20,000 g at 8°C, the His-tagged recombinant proteins were loaded on to a Ni-NTA column (Qiagen Inc.), and eluted with imidazole in buffer A. The protein was then loaded onto a Superdex TM200 column (GE), equilibrated in 100 mM NaCl, 1 mM ATP, 3 mM MgCl_2_, 20 mM Tris-HCl (pH 7.5). The protein was concentrated using Vivaspin 5,000 nominal molecular weight limit cut-off centrifugal concentrators (Vivascience), aliquoted and flash frozen in liquid nitrogen, and stored at −80°C.

### DNA-Binding Assays

Fluorescence anisotropy was measured in a CARY Eclipse (Varian) spectrofluorometer, at 20°C, in a final reaction volume of 200 μL buffered with 25 mM NaCl, 20 mM Tris (pH 7.5), 2.5 mM MgCl_2_, 2.5% (vol/vol) glycerol, and supplemented with 25 nM of a 20-mers, 50-mers, or 100-mers of a polydT oligonucleotide, or 35-mers oligonucleotides of various GC content percentage, and then 5′-labeled with fluoresceine (GeneCust). The excitation wavelength was set to 490 nm, and emission was observed at 525 nm (10 nm bandwidth). Protein injections were 0.25–1 μL from a 10 mg/mL stock solution. The data were treated with SigmaPlot 12.0 (Systat Software).

### Structural Modeling of DprA

A structural model of the full-length protein was made using the Swiss Model server (Schwede et al., 2003) and the crystal structure of DprA from *R. palustris* as a template (PDB ID = 3MAJ).

### Cloning and Yeast Two-Hybrid (Y2H) Assays

Yeast strain pJ69-4A was the host for the Y2H experiments, using plasmids pGAD-C1 and pGBDU-C1 as the starting material for generating plasmids encoding Gal4-AD and Gal4-BD fusion proteins, respectively (James et al., 1996). *D. radiodurans dprA, recA, recO*, and *ddrB* genes were subcloned into plasmid pGAD-C1 as well as into plasmid pGBDU-C1 for *dprA, recA* and *ddrB* in order to test for cross-interactions using Y2H assays. Two versions, with varying length of the N-terminal α-helix of RecA (full-length FL and ΔN27), were constructed. Pairs of pGAD-C1 and pGBDU-C1 variants were introduced into strain pJ69-4A by co-transformation, and transformants were selected on synthetic complete medium lacking leucine and uracil. Interactions between AD and BD fusions were assayed for selected clones on synthetic complete medium lacking leucine, uracil, and histidine. The clones were spotted as a series of 1/5 dilutions.

## Data Availability Statement

The raw data supporting the conclusions of this article will be made available by the authors, without undue reservation, to any qualified researcher.

## Author Contributions

SI, GC, JL, DL, NE, EB, CB, and PS: Performing the experiments. PS and SQ-C: Conceptualization, supervision, and writing—original draft preparation. SI, SS, GC, PS, JL, and SQ-C: Analyzing data. SS, EB, CB, SQ-C, FC, SI, and PS: Writing—review and editing.

## Conflict of Interest

The authors declare that the research was conducted in the absence of any commercial or financial relationships that could be construed as a potential conflict of interest.
