# Supplementary material for: Natural Transformation in Deinococcus radiodurans: A Genetic Analysis Reveals the Major Roles of DprA, DdrB, RecA, RecF, and RecO Proteins
Source: Front Microbiol. 2020 Jun 18;11:1253. doi: 10.3389/fmicb.2020.01253 (PMC7314969; doi:10.3389/fmicb.2020.01253)
Supplement: Supplementary file 1 [file Table_1.docx]

| **strains** | **genotype** | **Generation time (min)** |
| --- | --- | --- |
| GY 9613 | wild type | 91 (+/- 4) |
| GY 12835 | ∆*ddrB*Ω*kan* | 88 (+/-3) |
| GY 15121 | ∆*dprA*Ω*kan* | 87 (+/-4) |
| GY 16683 | Δ*dr1854*-*dr1855*Ω(*comEC*/*comEA*)Ω*kan* | 92 (+/- 3) |
| GY 17018 | Δ*dr0774(pilQ)*Ω*cat* | 90 (+/-4) |
| GY 17019 | ∆*dr0207(comEA)*Ω*cat* | 88 (+/-4) |
| GY 17020 | ∆*dr2065(pilD)*Ω*cat* | 87 (+/-3) |
| GY 17021 | Δ*fimA*Ω*cat* | 90 (+/-4) |
| GY 17782 | Δ*dr1232(pilIV)*Ω*cat* | 94 (+/-4) |
| GY 17784 | Δ*dr0548(pilIV)*Ω*kan* | 97 (+/- 2) |
| GY 17786 | Δ*dr0847(comA)*Ω*cat* | 93 (+/- 2) |
| GY 17788 | Δ*dr1389(comF)*Ω*kan* | 92 (+/- 2) |
| GY 17790 | Δ*dr1963*(*pilT*)Ω*cat* | 94 (+/- 2) |
| GY 17792 | Δ*dr1964*(*pilB*)Ω*cat* | 93 (+/- 3) |
| GY 17796 | Δ*dr0361(comEC)*Ω*kan* | 89 (+/-3) |

**Table S1:** generation time of different mutants. The strains were grown at 30°C in TGY2X medium. The A_650_ nm values of the culture were measured and generation times calculated from 4 independent experiments.
