# Supplementary material for: Natural Transformation in Deinococcus radiodurans: A Genetic Analysis Reveals the Major Roles of DprA, DdrB, RecA, RecF, and RecO Proteins
Source: Front Microbiol. 2020 Jun 18;11:1253. doi: 10.3389/fmicb.2020.01253 (PMC7314969; doi:10.3389/fmicb.2020.01253)
Supplement: Supplementary file 2 [file Table_2.docx]

**Table S2.** Overview of primers used for construction of mutant strains, cloning and diagnostic pcr experiments.

| Primer | Primer sequence 5’ 3’ | Use |
| --- | --- | --- |
| **Gene deletion** | | |
|  |  |  |
| ***dprA dprA* deletion (tripartite ligation procedure, strain GY15121)** | |  |
| EB109 | ATTT**GGATCC**GGGTCACGGCGCGAGAATAG | Amplification of *dprA* upstream region (c) |
| EB110 | TGAGAGCCGTCCAGACCTTC | Amplification of *dprA* upstream region |
| EB111 | ATTA**TCTAGA**CGGGCGTTGGAGTCGCTGAG | Amplification of *dprA* downstream region |
| EB112 | TGGGTGATCTTGAGGATGAC | Amplification of *dprA* downstream region (c) |
| Kan5Bam | GGAA**GGATCC**GCATTCTGCCTCCAGCATCTC | Amplification of a *kan* cassette |
| Kan3Xba | GGAA**TCTAGA**GCAAGCAGCAGATTACG | Amplification of a *kan* cassette (c) |
|  |  |  |
| ***recO* deletion (tripartite ligation procedure, strain GY16052)** | |  |
| EB73 | ACGACCACGCGGTACATCTG | Amplification of *recO* upstream region |
| EB74 | AAT**GGATCC**TGACGATGCCGCTGCGGTTG | Amplification of *recO* upstream region (c) |
| EB75 | ATA**TCTAGA**GCGGCAACTCGTCCCAAGCG | Amplification of *recO* downstream region |
| EB76 | CGTTGCTGACGCCTCAGAAG | Amplification of *recO* downstream region (c) |
| PS39 | ACG**GGATCC**CTTTGGAACGGTGCTCGGTG | Amplification of a *cat* cassette |
| PS178 | ATT**TCTAGA**CGCGGCCGCACTTATTCA | Amplification of a *cat* cassette (c) |
|  |  |  |
| ***dr1854*-*dr1855*Ω*kan* deletion (tripartite ligation procedure, strain GY16683)** | | |
| PS678 | ACATAGTTCGCCAGCATCCG | Amplification of *dr1854-dr1855* upstream region |
| PS679 | ATCT**GGATCC**CGTTCGTGAGGGAGCATGTG | Amplification of *dr1854-dr1855* upstream region (c) |
| PS680 | ATTG**TCTAGA**AGGTATGGCGGACCGATCAG | Amplification of *dr1854-dr1855* downstream region |
| PS681 | GCCTTTGCGTTCTGCGTTTC | Amplification of *dr1854-dr1855* downstream region (c) |
| Kan5Bam | GGAA**GGATCC**GCATTCTGCCTCCAGCATCTC | Amplification of a *kan* cassette |
| Kan3Xba | GGAA**TCTAGA**GCAAGCAGCAGATTACG | Amplification of a *kan* cassette (c) |
|  |  |  |
| ***dr0774* (*pilQ*) deletion (tripartite ligation procedure, strain GY 17018)** | | |
| GC7 | GGTCAAAGGCCTAAGCAGTTGG | Amplification of *pilQ* upstream region |
| GC8 | GACC**GGATCC**TGTAGATGCGCTGCACAATAG | Amplification of *pilQ* upstream region (c) |
| GC9 | ATGG**TCTAGA**TCATCACCGGCACCGTCGTC | Amplification of *pilQ* downstream region |
| GC10 | TTCTGAATGGCGAGGGTGAC | Amplification of *pilQ* downstream region (c) |
| PS177 | ATT**GGATCC**TATACGGAACCTATACGGG | Amplification of a *cat* cassette |
| PS178 | ATT**TCTAGA**CGCGGCCGCACTTATTCA | Amplification of a *cat* cassette (c) |
|  |  |  |
| ***dr0548* deletion** (**tripartite ligation procedure, strain GY 17784)** | | |
| SI283 | ATCT**GGATCC**TCATGGTGAATCTCCTGTGG | Amplification of *dr0548* upstream region (c) |
| SI284 | CTGCCCAGCACGCTGATTTC | Amplification of *dr0548* upstream region |
| SI285 | CTGG**TCTAGA**GGTACCGGCAGCAACTAATC | Amplification of *dr0548* downstream region |
| SI286 | TCCGGCGATACTCTTGGTGC | Amplification of *dr0548* downstream region (c) |
| Kan5Bam | GGAA**GGATCC**GCATTCTGCCTCCAGCATCTC | Amplification of a *kan* cassette |
| Kan3Xba | GGAA**TCTAGA**GCAAGCAGCAGATTACG | Amplification of a *kan* cassette (c) |
|  |  |  |
| ***dr1389* deletion (tripartite ligation procedure, strain GY 17788)** | | |
| SI295 | TCA**GGATCC**ATCATTGGCCCGGAGTGTAG | Amplification of *dr1389* upstream region (c) |
| SI296 | CGCCAATCAGGTGAGCTACG | Amplification of *dr1389* upstream region |
| SI297 | AGT**TCTAGA**CTGAAATACGCGGCGGTAGC | Amplification of *dr1389* downstream region |
| SI298 | TGGATGCTCTGGCTGATTTC | Amplification of *dr1389* downstream region (c) |
| Kan5Bam | GGAA**GGATCC**GCATTCTGCCTCCAGCATCTC | Amplification of a *kan* cassette |
| Kan3Xba | GGAA**TCTAGA**GCAAGCAGCAGATTACG | Amplification of a *kan* cassette (c) |
|  |  |  |
| ***dr0207* (*comEA*) deletion (tripartite ligation procedure, strain GY 17019)** | | |
| GC15 | TGATGTATGGCGCGGACACG | Amplification of *dr0207* upstream region |
| GC16 | ATCT**GGATCC**GCAGGGTCTTGGTCATAAGC | Amplification of *dr0207* upstream region (c) |
| GC17 | ATGC**TCTAGA**CCTCGCCTTCTGAAGATTGC | Amplification of *dr0207* downstream region |
| GC18 | GACCGGCAAGCCCAAGAAAC | Amplification of *dr0207* downstream region (c) |
| PS177 | ATT**GGATCC**TATACGGAACCTATACGGG | Amplification of a *cat* cassette |
| PS178 | ATT**TCTAGA**CGCGGCCGCACTTATTCA | Amplification of a *cat* cassette (c) |
|  |  |  |
| ***dr2065* (*pilD*) deletion (tripartite ligation procedure, strain GY 17020)** | | |
| GC21 | ACGCGAGGACTGAGCGAAAG | Amplification of *dr2065* upstream region |
| GC22 | ATT**GGATCC**CGGCGAAGAGGACGATGAGGGTG | Amplification of *dr2065* upstream region (c) |
| GC23 | ATG**TCTAGA**GGGCTGCTAGGGATGTAACG | Amplification of *dr2065* downstream region |
| GC24 | TATGGAAACCGGCGGGATGC | Amplification of *dr2065* downstream region (c) |
| PS177 | ATT**GGATCC**TATACGGAACCTATACGGG | Amplification of a *cat* cassette |
| PS178 | ATT**TCTAGA**CGCGGCCGCACTTATTCA | Amplification of a *cat* cassette (c) |
|  |  |  |
| ***dr0361* (*comEC*) deletion (tripartite ligation procedure, strain GY 17796)** | | |
| PS805 | ATT**GGATCC**CATGATGTCCGCGCTGCTCG | Amplification of *dr0361* upstream region (c) |
| PS806 | TGGTGGACGGGCTGGAAGAC | Amplification of *dr0361* upstream region |
| PS807 | ATCG**TCTAGA**GGCGGACGGTTCCTACGTGG | Amplification of *dr0361* downstream region |
| PS808 | TGAGCTTCGACGCCGACTTC | Amplification of *dr0361* downstream region (c) |
| Kan5Bam | GGAA**GGATCC**GCATTCTGCCTCCAGCATCTC | Amplification of a *kan* cassette |
| Kan3Xba | GGAA**TCTAGA**GCAAGCAGCAGATTACG | Amplification of a *kan* cassette (c) |
|  |  |  |
| ***dr1232* deletion** (**tripartite ligation procedure, strain GY 17782)** | | |
| SI277 | TCA**GGATCC**CCGATTGTCCACGATCTTTC | Amplification of *dr1232* upstream region (c) |
| SI278 | CGACATCGGCATCGCTTCTG | Amplification of *dr1232* upstream region |
| SI279 | ATAG**TCTAGA**TGGGCGTAACCGGCAAAGTC | Amplification of *dr1232* downtream region |
| SI280 | CATGGCAGGCACCGACAAAG | Amplification of *dr1232* downtream region (c) |
| PS177 | ATT**GGATCC**TATACGGAACCTATACGGG | Amplification of a *cat* cassette |
| PS178 | ATT**TCTAGA**CGCGGCCGCACTTATTCA | Amplification of a *cat* cassette (c) |
|  |  |  |
| ***dr0847* deletion (tripartite ligation procedure, strain GY 17786)** | | |
| SI289 | ATT**GGATCC**TGGGGCCACTGTAGAGGGTC | Amplification of *dr0847* upstream region (c) |
| SI290 | CAGCAAAGTGCTGCAAGACC | Amplification of *dr0847* upstream region |
| SI291 | ATCG**TCTAGA**AACGTGATGGCGCTGTTCCG | Amplification of *dr0847 d*owntream region |
| SI292 | GCCACGACTTGACGAGTTTG | Amplification of *dr0847* downtream region (c) |
| PS177 | ATT**GGATCC**TATACGGAACCTATACGGG | Amplification of a *cat* cassette |
| PS178 | ATT**TCTAGA**CGCGGCCGCACTTATTCA | Amplification of a *cat* cassette (c) |
|  |  |  |
| ***fimA* deletion (tripartite ligation procedure, strain GY 17021)** | | |
| GC27 | ATC**GGATCC**CCCACCGCGAGGAACTTGCTC | Amplification of *fimA* upstream region (c) |
| GC28 | GAAACGGGTTGACGGTGAGG | Amplification of *fimA* upstream region |
| GC29 | ATC**TCTAGA**CCGGCGGCGCTCGCCTATTTG | Amplification of *fimA* downstream region (c) |
| GC30 | CAGGCAGTGACCGTAGCTCAC | Amplification of *fimA* downstream region |
| PS177 | ATT**GGATCC**TATACGGAACCTATACGGG | Amplification of a *cat* cassette |
| PS178 | ATT**TCTAGA**CGCGGCCGCACTTATTCA | Amplification of a *cat* cassette (c) |
|  |  |  |
| ***dr1963* (*pilT*) deletion (tripartite ligation procedure, strain GY17790)** | | |
| PS793 | ATC**CTCGAG**TGGTCATGGGTCAGCTTTCC | Amplification of *pilT* upstream region (c) |
| PS794 | TGACCCGCCTTGAGGAAATG | Amplification of *pilT* upstream region |
| PS795 | CTCGAAAGCCGCGTGAGTAG | Amplification of *pilT* downstream region (c) |
| PS796 | ATT**TCTAGA**GGACGCGCCGGAGGCTTTGG | Amplification of *pilT* downstream region |
| FV95 | ATC**CTCGAG**CGGAACCTATACGGGAACTG | Amplification of a *cat* cassette |
| PS178 | ATT**TCTAGA**CGCGGCCGCACTTATTCA | Amplification of a *cat* cassette (c) |
|  |  |  |
| ***dr1964* (*pilB*) deletion (tripartite ligation procedure, strain GY17792)** | | |
| PS799 | ATC**CTCGAG**CGGCGGTCACCAATCGAAAG | Amplification of *pilB* upstream region (c) |
| PS800 | CTTCTGGCTGCCGAAACTGG | Amplification of *pilB* upstream region |
| PS801 | GCC**TCTAGA**ACCACCCTTGAAGAAGTGCTG | Amplification of *pilB* downstream region (c) |
| PS802 | AGGCGAGGGTGGTCGATTTG | Amplification of *pilB* downstream region |
| FV95 | ATC**CTCGAG**CGGAACCTATACGGGAACTG | Amplification of a *cat* cassette |
| PS178 | ATT**TCTAGA**CGCGGCCGCACTTATTCA | Amplification of a *cat* cassette (c) |
|  |  |  |
| ***amyE* deletion (tripartite ligation procedure, strains GY 17140 and GY 17130)** | | |
| HH47 | ATT**GGATCC**GGGGGAGATGCTAGCGTTCG | Amplification of *amyE* upstream region (c) |
| HH48 | AACGGGTAAACGCCGCCTTC | Amplification of *amyE* upstream region |
| HH49 | TGA**TCTAGA**GAGGAGGAGTCTGACTTG | Amplification of *amyE* downstream region |
| HH50 | CGGCCTTCTTCTCGTACTCG | Amplification of *amyE* downstream region (c) |
| PS177 | ATT**GGATCC**TATACGGAACCTATACGGG | Amplification of a *cat* cassette |
| PS178 | ATT**TCTAGA**CGCGGCCGCACTTATTCA | Amplification of a *cat* cassette (c) |
| Kan5Bam | GGAA**GGATCC**GCATTCTGCCTCCAGCATCTC | Amplification of a *kan* cassette |
| Kan3Xba | GGAA**TCTAGA**GCAAGCAGCAGATTACG | Amplification of a *kan* cassette (c) |
|  |  |  |
| **Strains with HA tag** | | |
|  |  |  |
| ***dprA*::HAΩ*kan* (tripartite ligation procedure, strain GY16054)** | | |
| SI60 | ATTC**GGATCC**GCGACTCCAACGCCCGCCCA | Amplification of *dprA* region |
| SI61 | GGACGTGATTTATCCGCGTG | Amplification of *dprA* region (c) |
| EB111 | ATTA**TCTAGA**CGGGCGTTGGAGTCGCTGAG | Amplification of *dprA* downstream region |
| EB112 | TGGGTGATCTTGAGGATGAC | Amplification of *dprA* downstream region (c) |
| PS455 | GGACC**GGATCC**TACCCGTACGACGTGCCCGAC | Amplification of Ha-tag kanamycine cassette |
| PS457 | AAAT**TCTAGA**CGAATTGGGCCCGGTCTGAC | Amplification of Ha-tag kanamycine cassette (c) |
|  |  |  |
| **∆Nter *dprA*Ωcat (quatuor ligation procedure, strain GY16056)** | | |
| SI73 | CAAGACGCTGAACTACTTCG | Amplification of *dr0121* upstream region |
| SI72-*BamHI* | TCAC**GGATCC**CTAGCTTCAATCGGAATTCG | Amplification of *dr0121* upstream region (c) |
| PS39-*BamHI* | ACG**GGATCC**CTTTGGAACGGTGCTCGGTG | Amplification of a *cat* cassette |
| PS178-*XbaI* | ATT**TCTAGA**CGCGGCCGCACTTATTCA | Amplification of a *cat* cassette (c) |
| SI75-*XbaI* | ACTG**TCTAGA**TGTTTTGGCTTCAATCCAAG | Amplification of P*_dprA_* |
| SI59-*BsrDI* | GCAGCT**CCATTGCG**CGAGAATAGGCGGGGCG | Amplification of P*_dprA_* (c) |
| SI58-*BsrDI* | TCAT**GCAATGGA**GCGCGGCGTCACCCTGCT | Amplification of **∆**Nter *dprA* |
| SI76 | TGCACTTTCGTGCCGTTTCG | Amplification of **∆**Nter *dprA* (c) |
|  |  |  |
| **∆Nter *dprA::HA*Ω*cat* (tripartite ligation procedure, strain GY16201)** | | |
| SI60 | ATTC**GGATCC**GCGACTCCAACGCCCGCCCA | Amplification of *dprA* region |
| SI61 | GGACGTGATTTATCCGCGTG | Amplification of *dprA* region (c) |
| SI56 | GCTA**TCTAGA**GCGGGGTCTTTTGCTAGACT | Amplification of *dprA* downstream region |
| EB112 | TGGGTGATCTTGAGGATGAC | Amplification of *dprA* downstream region (c) |
| PS455 | GGACC**GGATCC**TACCCGTACGACGTGCCCGAC | Amplification of Ha-tag kanamycine cassette |
| PS457 | AAAT**TCTAGA**CGAATTGGGCCCGGTCTGAC | Amplification of Ha-tag kanamycine cassette (c) |
|  |  |  |
| ***dprA*∆CterΩ*cat* (tripartite ligation procedure, strain GY 16058)** | | |
| SI54 | TCAT**GGATCC**TCACCAGTTCAGCTCGGTCAGCA | Amplification of *drpA* region (c) |
| SI55 | ACTGAACAAGGCGGCAGAGC | Amplification of *drpA* region |
| PS39 | ACG**GGATCC**CTTTGGAACGGTGCTCGGTG | Amplification of a *cat* cassette |
| PS178 | ATT**TCTAGA**CGCGGCCGCACTTATTCA | Amplification of a *cat* cassette (c) |
| SI56 | GCTA**TCTAGA**GCGGGGTCTTTTGCTAGACT | Amplification of *dprA* downstream region |
| EB112 | TGGGTGATCTTGAGGATGAC | Amplification of *dprA* downstream region (c) |
|  |  |  |
| ***dprA*∆Cter ::HAΩ*kan* (tripartite ligation procedure, strain GY 16205)** | | |
| SI108 | ATGC**GGATCC**CCAGTTCAGCTCGGTCAGCA | Amplification of *drpA* region (c) |
| SI55 | ACTGAACAAGGCGGCAGAGC | Amplification of *drpA* region |
| SI56 | GCTA**TCTAGA**GCGGGGTCTTTTGCTAGACT | Amplification of *dprA* downstream region |
| EB112 | TGGGTGATCTTGAGGATGAC | Amplification of *dprA* downstream region (c) |
| PS455 | GGACC**GGATCC**TACCCGTACGACGTGCCCGAC | Amplification of Ha-tag kanamycine cassette |
| PS457 | AAAT**TCTAGA**CGAATTGGGCCCGGTCTGAC | Amplification of Ha-tag kanamycine cassette (c) |
|  |  |  |
| **INSERTION OF GENES IN *amyE* LOCI** | | |
|  | | |
| ***dr2065* in *amyE* (tripartite ligation procedure, strain GY 17144)** | | |
| PS222 | GGCCTTCGAACGGGTAAACG | Amplification of *amyE* upstream region |
| EB137 | GGAA**CTCGAG**CATGGGGGAGATGCTAGCG | Amplification of *amyE* upstream region (c) |
| EB144 | GGAA**CTCGAG**ATTCTTCCCTGCTGCTCCTG | Amplification of P*_dr2065_*-*dr2065* |
| EB145 | GGACC**GGATCC**CTTTATCGCCGTTACATCCC | Amplification of P*_dr2065_*-*dr2065* (c) |
| Kan5Bam | GGAA**GGATCC**GCATTCTGCCTCCAGCATCTC | Amplification of a *kan*-*amyE* region |
| HH50 | CGGCCTTCTTCTCGTACTCG | Amplification of *kan*-*amyE* region (c) |
|  |  |  |
| ***dr1389* in *amyE* (tripartite ligation procedure, strain GY17138)** | | |
| PS222 | GGCCTTCGAACGGGTAAACG | Amplification of *amyE* upstream region |
| NE184 | TCA**CATATG**AGCAGCGCCGGGAGGATGAG | Amplification of *amyE* upstream region (c) |
| NE191 | TCA**CATATG**GACCTGACGCGCTTTAGCCC | Amplification of P*_dr1389_*-*dr1389* |
| NE192 | TCA**GGATCC**GCCCTGAACAGGCAGACTTAG | Amplification of P*_dr1389_*-*dr1389* (c) |
| PS177 | ATT**GGATCC**TATACGGAACCTATACGGG | Amplification of a *cat-amyE* region |
| HH50 | CGGCCTTCTTCTCGTACTCG | Amplification of *cat*-*amyE* region (c) |
|  |  |  |
| ***dprA* in *amyE* (tripartite ligation procedure, strain GY17136)** | | |
| PS222 | GGCCTTCGAACGGGTAAACG | Amplification of *amyE* upstream region |
| EB148 | AAAT**TCTAGA**GGGGGAGATGCTAGCGTTCG | Amplification of *amyE* upstream region (c) |
| SI75 | ACTG**TCTAGA**TGTTTTGGCTTCAATCCAAG | Amplification of P*_dprA_*-*dprA* |
| SI57 | ATCT**GGATCC**CGCTCAGCGACTCCAACGCC | Amplification of P*_dprA_*-*dprA* (c) |
| PS177 | ATT**GGATCC**TATACGGAACCTATACGGG | Amplification of a *cat-amyE* region |
| HH50 | CGGCCTTCTTCTCGTACTCG | Amplification of *cat*-*amyE* region (c) |
|  |  |  |
| ***dr0548* in *amyE* (tripartite ligation procedure, strain GY17132)** | | |
| PS222 | GGCCTTCGAACGGGTAAACG | Amplification of *amyE* upstream region |
| EB137 | GGAA**CTCGAG**CATGGGGGAGATGCTAGCG | Amplification of *amyE* upstream region (c) |
| EB138 | GGAA**CTCGAG**AAAGGCTCAAGCGCGTCCAG | Amplification of P*_dr0548_*-*dr0548* |
| EB139 | GGACC**GGATCC**CGGAGGGCGGTTCTGAGAAGAG | Amplification of P*_dr0548_*-*dr0548* (c) |
| PS177 | ATT**GGATCC**TATACGGAACCTATACGGG | Amplification of a *cat-amyE* region |
| HH50 | CGGCCTTCTTCTCGTACTCG | Amplification of *cat*-*amyE* region (c) |
|  |  |  |
| ***dr1854-dr1855* in *amyE* (tripartite ligation procedure, strain GY17134)** | | |
| PS222 | GGCCTTCGAACGGGTAAACG | Amplification of *amyE* upstream region |
| EB137 | GGAA**CTCGAG**CATGGGGGAGATGCTAGCG | Amplification of *amyE* upstream region (c) |
| EB140 | GGAA**CTCGAG**CATTCATCGCCCTGCTCCTT | Amplification of P*_dr1854-_dr1854-dr1855* |
| EB141 | GGAA**CTCGAG**CATTCATCGCCCTGCTCCTT | Amplification of P*_dr1854-_dr1854-dr1855* (c) |
| PS177 | ATT**GGATCC**TATACGGAACCTATACGGG | Amplification of a *cat-amyE* region |
| HH50 | CGGCCTTCTTCTCGTACTCG | Amplification of *cat*-*amyE* region (c) |
|  |  |  |
| **P*spac*-*recA* in *amyE* (tripartite ligation procedure, strain GY17154)** | | |
| PS222 | GGCCTTCGAACGGGTAAACG | Amplification of *amyE* upstream region |
| PS824 | ATT**GGTACC**TGGGGGAGATGCTAGCGTTC | Amplification of *amyE* upstream region (c) |
| PS823 | ATT**GGTACC**TTGAGGTAGCCCTTGCCTAC | Amplification of P*_spac_*-*recA* |
| NE189 | TCA**GGATCC**ACTGGAAAGCGGGCAGTGAG | Amplification of P*_spac_*-*recA* (c) |
| PS177 | ATT**GGATCC**TATACGGAACCTATACGGG | Amplification of a *cat-amyE* region |
| HH50 | CGGCCTTCTTCTCGTACTCG | Amplification of *cat*-*amyE* region (c) |
|  |  |  |
| **P*_recF_*-*recF* in *amyE* (tripartite ligation procedure, strain GY 18091)** | | |
| PS222 | GGCCTTCGAACGGGTAAACG | Amplification of *amyE* upstream region |
| EB148 | AAAT**TCTAGA**GGGGGAGATGCTAGCGTTCG | Amplification of *amyE* upstream region (c) |
| NE209 | TCA**TCTAGA**AGAGGCAGCTCCTTTCTCCAG | Amplification of P*_recF_*-*recF* |
| PS411 | ATCA**GGATCC**CATGTCGAGCGGGCCACTGC | Amplification of P*_recF_*-*recF* (c) |
| Kan5Bam | GGAA**GGATCC**GCATTCTGCCTCCAGCATCTC | Amplification of a *kan*-*amyE* region |
| HH50 | CGGCCTTCTTCTCGTACTCG | Amplification of *kan*-*amyE* region (c) |
|  |  |  |
| **P*_recO_*-*recO* in *amyE* (tripartite ligation procedure, strain GY 17148)** | | |
| PS222 | GGCCTTCGAACGGGTAAACG | Amplification of *amyE* upstream region |
| NE184 | TCA**CATATG**AGCAGCGCCGGGAGGATGAG | Amplification of *amyE* upstream region (c) |
| NE185 | TCA**CATATG**GATTTCGCGGTACGACTCGG | Amplification of P*_recO_*-*recO* |
| PS421 | ATTTA**GGATCC**GCTCAGCACCGGCACGCCGCTTG | Amplification of P*_recO_*-*recO* (c) |
| Kan5Bam | GGAA**GGATCC**GCATTCTGCCTCCAGCATCTC | Amplification of a *kan*-*amyE* region |
| HH50 | CGGCCTTCTTCTCGTACTCG | Amplification of *kan*-*amyE* region (c) |
|  |  |  |
| **P*_ddrB_*-*ddrB* in *amyE* (tripartite ligation procedure, strain GY 18099)** | | |
| PS222 | GGCCTTCGAACGGGTAAACG | Amplification of *amyE* upstream region |
| EB148 | AAAT**TCTAGA**GGGGGAGATGCTAGCGTTCG | Amplification of *amyE* upstream region (c) |
| PS523 | ATCA**GGATCC**AAAGCAGGCCGCCTTCCTTC | Amplification of P*_ddrB_*-*ddrB* (c) |
| PS524 | ATGG**TCTAGA**GGCGCCGCTAAGACCACCTG | Amplification of P*_ddrB_*-*ddrB* |
| Kan5Bam | GGAA**GGATCC**GCATTCTGCCTCCAGCATCTC | Amplification of a *kan*-*amyE* region |
| HH50 | CGGCCTTCTTCTCGTACTCG | Amplification of *kan*-*amyE* region (c) |
|  |  |  |
| **P*_dr1964_*-*dr1964* in *amyE* (tripartite ligation procedure, strain GY 17152)** | | |
| PS222 | GGCCTTCGAACGGGTAAACG | Amplification of *amyE* upstream region |
| EB148 | AAAT**TCTAGA**GGGGGAGATGCTAGCGTTCG | Amplification of *amyE* upstream region (c) |
| EB146 | AAAT**TCTAGA**GTAAGCTAGCGCCACCTCCC | Amplification of P*_dr1964_*-*dr1964* |
| EB147 | TAAT**GGTACC**CTTCTCTGGACGCGGCAGGC | Amplification of P*_dr1964_*-*dr1964* (c) |
| SI169 | TGAT**GGTACC**GCATTCTGCTCCAGCATCTC | Amplification of a *kan*-*amyE* region |
| HH50 | CGGCCTTCTTCTCGTACTCG | Amplification of *kan*-*amyE* region (c) |
|  |  |  |
| **P*_dr1963_*-*dr1963* in *amyE* (tripartite ligation procedure, strain GY 16964)** | | |
| PS222 | GGCCTTCGAACGGGTAAACG | Amplification of *amyE* upstream region |
| EB148 | AAAT**TCTAGA**GGGGGAGATGCTAGCGTTCG | Amplification of *amyE* upstream region (c) |
| PS836 | ATCT**GGTACC**GTGCAGGCGGCGTTTTATTG | Amplification of P*_dr1963_*-*dr1963* |
| PS837 | ATCG**TCTAGA**AGAAGTGCTGGCCGTCACCG | Amplification of P*_dr1963_*-*dr1963* (c) |
| SI169 | TGAT**GGTACC**GCATTCTGCTCCAGCATCTC | Amplification of a *kan*-*amyE* region |
| HH50 | CGGCCTTCTTCTCGTACTCG | Amplification of *kan*-*amyE* region (c) |
|  |  |  |
| **DIAGNOSTIC PRIMERS** | | |
|  |  |  |
| ***dprA* deletion, *dprA*::HAΩ*kan, ∆*Nter *dprA*Ωcat, *dprA*∆CterΩ*cat, dprA*∆Cter ::HAΩ*kan,* ∆Nter *dprA::HA*Ω*cat*** | | |
| EB112 | TGGGTGATCTTGAGGATGAC | Verification of *dprA* construction (c) |
| EB113 | ACAGTGACCGCTACAACCTC | Verification of *dprA* construction |
|  |  |  |
| ***recO* deletion** | | |
| PS420 | TGAAACAGGCGGTGCTGGAG | Test for absence of wild type *recO* allele |
| EB76 | CGTTGCTGACGCCTCAGAAG | Test for absence of wild type *recO* allele (c) |
| PS94 | TTAAACGTGGCCAATATGGACAACT | Verification of right junction *recO* deletion |
| EB84 | TTTGTGTTGGCCTTCCTCAG | Verification of right junction *recO* deletion (c) |
|  |  |  |
| ***recA* deletion** |  |  |
| EB91 | TTCTCCTCGAAGCCGACCTG | Verification of *recA* deletion |
| EB92 | CAGATGCACGTCACCCTCTC | Verification of *recA* deletion (c) |
|  |  |  |
| ***recF* deletion** |  |  |
| PS314 | CACGGCGCTGAACACTGCAC | Verification *recF* deletion |
| PS430 | AAACGCAGTTCGCTGACCTC | Verification *recF* deletion (c) |
| PS93 | TATCCAGCTGAACGGTCTGGTTA | Verification of right junction *recF* deletion with PS314 (c) |
|  |  |  |
| ***ddrB* deletion** |  |  |
| PS359 | ATGCGGGCGTTCGCAAGTCG | Verification of *ddrB* deletion |
| PS360 | ACCGCTTCTCGGTGGGCTA | Verification of *ddrB* deletion (c) |
|  | |  |
| ***dr0774* (*pilQ*) deletion** | |  |
| GC11 | CAACGAAGCTTGGCCTTTGC | Verification of *dr0774* deletion |
| GC12 | AGGCGACATGATTTCAGTCC | Verification of *dr0774* deletion (c) |
|  |  |  |
| ***dr0207* deletion** | | |
| GC19 | TGACCGCACCTGAAGATTCC | Verification of *dr0207* deletion |
| GC20 | AGCCGATGAACGTGAACTGG | Verification of *dr0207* deletion (c) |
|  |  |  |
| ***dr2065* deletion** | |  |
| GC25 | AGGAAAGCAGTCAGAGCAGTG | Verification of *dr2065* deletion |
| GC26 | GACTGAGCTGCTGAGCAACC | Verification of *dr2065* deletion (c) |
|  |  |  |
| ***fimA* deletion** | | |
| GC31 | AAAATCGCGCCGCCGTTCTC | Verification of *fimA* deletion |
| GC32 | TGAGACCCAGCGTAGTACAC | Verification of *fimA* deletion (c) |
|  |  |  |
| ***dr1854*-*dr1855* deletion** | | |
| PS682 | GCTTTGCCGACGATGACTGC | Verification of *dr1854-dr1855* deletion |
| PS683 | GTCTGGACGCTGGTAATTTC | Verification of *dr1854-dr1855* deletion (c) |
|  |  |  |
| ***dr0361* deletion** | | |
| PS809 | ACATGAACGAGCGAGAACAG | Verification of *dr0361* deletion |
| PS810 | AAGTGGGCATCCTCGGCAAC | Verification of *dr0361* deletion (c) |
|  |  |  |
| ***dr0548* deletion** | | |
| SI287 | CTCGCGGTAAAACATTTCGG | Verification of *dr0548* deletion |
| SI288 | TACGAACGGCACCGTGTTCC | Verification of *dr0548* deletion (c) |
|  |  |  |
| ***dr1232* deletion** | | |
| SI281 | GCTTCGTTTGCCAAGATCAC | Verification of *dr1232* deletion |
| SI282 | ACCTGGGTGCCGTTTGTTCC | Verification of *dr1232* deletion (c) |
|  |  |  |
| ***dr0847*deletion** | | |
| SI293 | AGCGGGTGCAGCACCTCAAC | Verification of *dr0847* deletion |
| SI294 | TGTCAATCGCGGCAGCAACC | Verification of *dr0847* deletion (c) |
|  |  |  |
| ***dr1389* deletion** | | |
| SI299 | CGACGGTCAAGACCAAGTTC | Verification of *dr1389* deletion |
| SI300 | GCCGCAAGGACGAGCATTTC | Verification of *dr1389* deletion (c) |
|  |  |  |
| ***dr1963* (*pilT*) deletion** | | |
| PS797 | ACATGGTGCTCGCCACTCTG | Verification of *pilT* deletion |
| PS798 | CAGCCGATGTACACGACCAC | Verification of *pilT* deletion (c) |
|  |  |  |
| ***dr1964* (*pilB*) deletion** | | |
| PS803 | GCCGCCGAAATAATCGCCTG | Verification of *pilB* deletion |
| PS804 | GCTTCTTGGTCGTGTTGATG | Verification of *pilB* deletion (c) |
|  |  |  |
| **insertion of *genes* in *amyE* loci** | | |
| PS216 | CGCCTCTGACAACCCTCCTG | Primer upstream of *amyE* gene |
| PS224 | ATTCTGCAGGCCGATGTAGCTCAGTG | Primer downstream of *amyE* gene (c) |
|  |  |  |
| **Clonage for two-hybrid assays** | | |
|  |  |  |
| ***recA*** |  |  |
| recAEco | TTTTT**GAATTC**AGCAAGGACGCCACCAAAGAAATCTCCGC | Amplification of *recA* |
| recA-DNA27 | TTTTT**gaattcc**agatcgaaaaggccttcggcaagggc | Amplification of ∆27*recA* |
| recA-pst | TTTTT**ctgcag**TTACGCTTCGGCGGCTTCGGGC | Amplification of *recA* (c) |
|  |  |  |
| ***recO*** |  |  |
| recO-Eco | TTTTT**gaattc**CGCTCACGCACCGCCAACCGCAGC | Amplification of *recO* |
| RecO-Pst | TTTTT**ctgcag**TTAGCTCAGCACCGGCACGCCGCT | Amplification of *recO* (c) |
|  |  |  |
| ***ddrB*** |  |  |
| ddrB-Eco | TTTTT**gaattc**TTGCAGATTGAATTTATCACCGAC | Amplification of *ddrB* |
| ddrB-Pst | TTTTT**ctgcag**TTAGAACGGCGTTTCTTCTTCCTGAC | Amplification of *ddrB* (c) |
|  |  |  |
| ***dprA*** |  |  |
| dprA-Eco | TTTTT**gaattc**ACCCTTCCCTCCCCTGCTGCCG | Amplification of *dprA* |
| dprA-Pst | TTTTT**ctgcag**TTAGCGACTCCAACGCCCGCCCACTT | Amplification of *dprA* (c) |
|  |  |  |
| **CLONAGE in pET29** | | |
| ***dprA*** |  |  |
| dprA-Nde | GGTC**CATATG**GTGACCCTTCCCTCCCCTGCT | Amplification of *dprA* |
| dprA-Xho | GGTC**CTCGAG**TTAATGGTGATGGTGATGGTGCCAGTTCAGCTCGGTCAGCAC | Amplification of *dprA* (c) |
|  |  |  |
