# Supplementary material for: Natural Transformation in Deinococcus radiodurans: A Genetic Analysis Reveals the Major Roles of DprA, DdrB, RecA, RecF, and RecO Proteins
Source: Front Microbiol. 2020 Jun 18;11:1253. doi: 10.3389/fmicb.2020.01253 (PMC7314969; doi:10.3389/fmicb.2020.01253)
Supplement: Supplementary file 3 [file Presentation_1.pptx]

## Slide 1
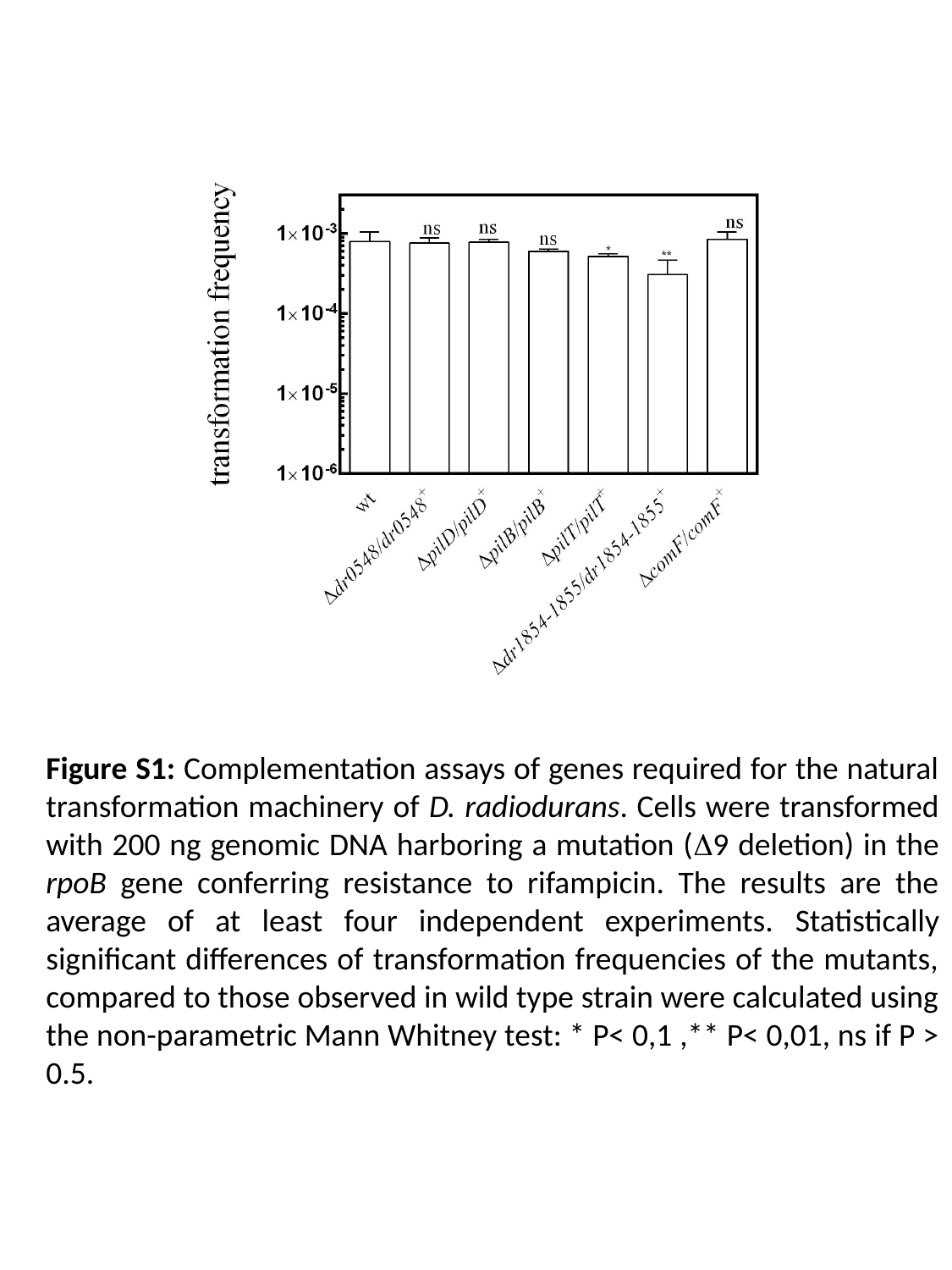

Figure S1: Complementation assays of genes required for the natural transformation machinery of D. radiodurans. Cells were transformed with 200 ng genomic DNA harboring a mutation (Δ9 deletion) in the rpoB gene conferring resistance to rifampicin. The results are the average of at least four independent experiments. Statistically significant differences of transformation frequencies of the mutants, compared to those observed in wild type strain were calculated using the non-parametric Mann Whitney test: * P< 0,1 ,** P< 0,01, ns if P > 0.5.

## Slide 2
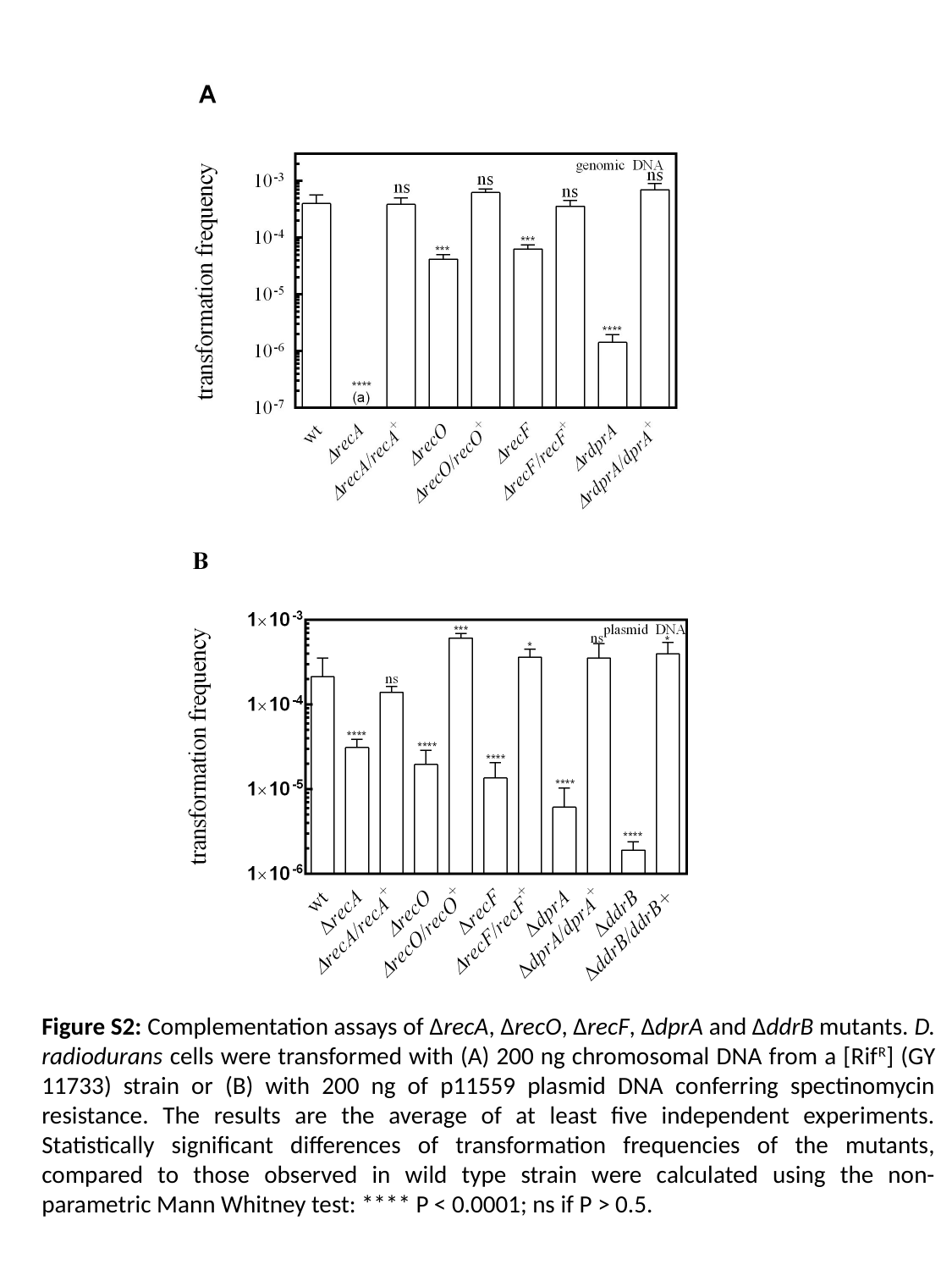

Figure S2: Complementation assays of ∆recA, ∆recO, ∆recF, ∆dprA and ∆ddrB mutants. D. radiodurans cells were transformed with (A) 200 ng chromosomal DNA from a [RifR] (GY 11733) strain or (B) with 200 ng of p11559 plasmid DNA conferring spectinomycin resistance. The results are the average of at least five independent experiments. Statistically significant differences of transformation frequencies of the mutants, compared to those observed in wild type strain were calculated using the non-parametric Mann Whitney test: **** P < 0.0001; ns if P > 0.5.

## Slide 3
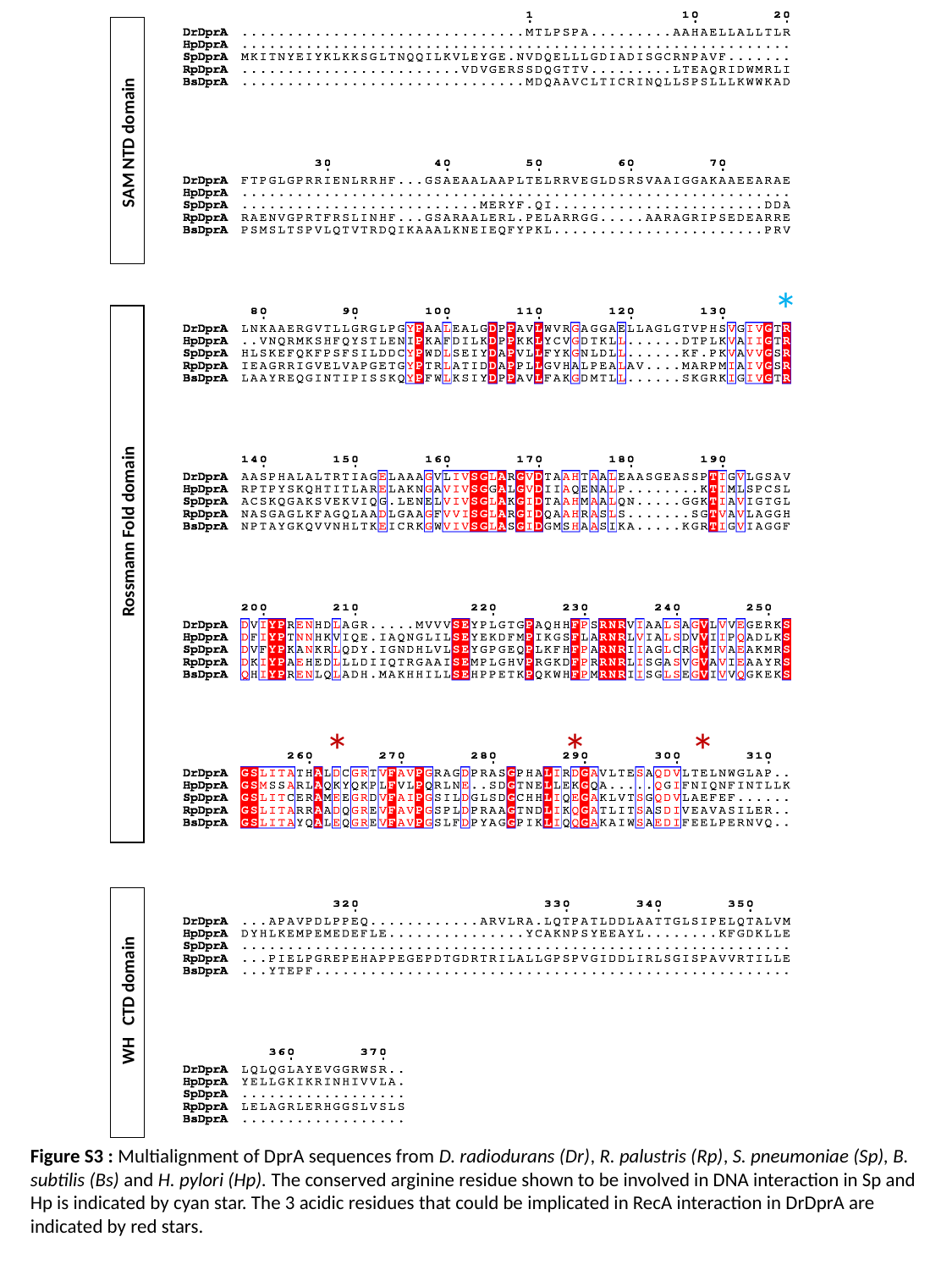

SAM NTD domain
*
 Rossmann Fold domain
*
*
*
 WH CTD domain
Figure S3 : Multialignment of DprA sequences from D. radiodurans (Dr), R. palustris (Rp), S. pneumoniae (Sp), B. subtilis (Bs) and H. pylori (Hp). The conserved arginine residue shown to be involved in DNA interaction in Sp and Hp is indicated by cyan star. The 3 acidic residues that could be implicated in RecA interaction in DrDprA are indicated by red stars.
